# Endocytome profiling uncovers cell-surface protein dynamics underlying neuronal connectivity

**DOI:** 10.1016/j.neuron.2026.01.027

**Published:** 2026-03-12

**Authors:** Colleen N. McLaughlin, Hui Ji, Katherine X. Dong, Chuanyun Xu, Kenneth Kin Lam Wong, Zhuoran Li, David J. Luginbuhl, Charles Xu, Cheng Lyu, Wei Qin, Jiefu Li, Namrata D. Udeshi, Steven A. Carr, Alice Y. Ting, Liqun Luo

**Affiliations:** 1 Department of Biology, Howard Hughes Medical Institute, Stanford University, Stanford, CA 94305, USA; 2 Broad Institute of MIT and Harvard, Cambridge, MA 02142, USA; 3 Department of Genetics, Biology, and Chemistry, Chan Zuckerberg Biohub, Stanford University, Stanford, CA 94305, USA; 4 Janelia Research Campus, Howard Hughes Medical Institute, Ashburn, VA 20147, USA; 5 Lead contact

## Abstract

Endocytosis actively remodels the neuronal surface proteome to drive diverse cellular processes, yet its global extent and effects on neural circuit development have defied comprehensive interrogation. Here, we introduce endocytome profiling: a systematic, cell-type-specific approach for mapping cell-surface protein (CSP) dynamics *in situ*. Quantitative proteomic analysis of developing *Drosophila* olfactory receptor neuron (ORN) axons generated an endocytic atlas comprising over 1,000 proteins and revealed the extent to which the cell-surface proteome is remodeled to meet developmental demands. Targeted interrogation of a junctional CSP showed that its endosome-to-surface ratio is precisely balanced to enable developmental axon pruning while preserving mature axon integrity. Multi-omic integration uncovered widespread transcellular signaling and identified a growth factor secreted by neighboring neurons to direct ORN axon targeting via endocytic regulation of its receptor. Endocytome profiling provides unprecedented access to cell-surface proteome dynamics and offers a platform to dissect proteome-scale remodeling across diverse cell types and contexts.

## In brief

Endocytome profiling is a powerful approach to decode rapid remodeling of the cell-surface proteome across cell states and contexts in intact tissues. Applying this tool to developing axons, McLaughlin et al. reveal how endocytosis enables neurons to reshape their plasma membrane protein composition to build and refine neural circuit connectivity.

## Graphical abstract

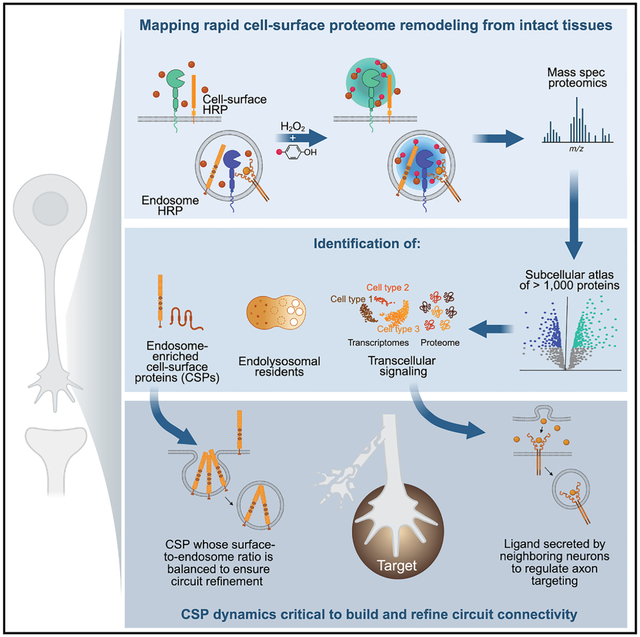

## INTRODUCTION

Neurons must rapidly adapt to evolving developmental signals and physiological needs. The plasma membrane is the interface between neurons and their environment. Here, cell-surface proteins (CSPs) transduce external cues into the intracellular responses that control neurodevelopment and physiology. Transcriptional changes^1–5^ govern extensive, long-timescale shifts in the CSP landscape observed between developing and mature neurons.^6,7^ However, neurodevelopmental processes, such as neurite growth and retraction, occur on much shorter timescales and thus require more rapid regulatory mechanisms. Targeted, on-demand surface proteome remodeling is well poised to shape these events. Although proteome-scale dynamics are increasingly appreciated in human cell lines,^8–10^ the degree and impact of rapid cell-surface remodeling in neurons or other cells *in vivo* are unknown.

Endocytosis actively sculpts the cell-surface proteome. Within minutes,^11^ soluble and membrane-anchored proteins are internalized from the plasma membrane into intracellular vesicles, which facilitate their degradation or redistribution throughout the cell. These trafficking events modulate CSP signaling and adhesive functions and, depending on the context, can lead to signal attenuation, reactivation, or engagement of new molecular partners.^12,13^ Mechanistic studies have elucidated the post-endocytic itineraries for select CSPs and revealed essential roles in processes ranging from neuronal survival^14^ to axon guidance^15,16^ and polarity.^17^ However, these examples represent only a small fraction of the neuronal surface proteome. Thus, the extent of CSP endocytosis and identity of internalized cargos remain unknown for most cell types and developmental contexts. This gap stems, in part, from a lack of tools for simultaneous, quantitative comparison of both plasma membrane and endosomal compartments within the same cell type and physiological state.

Here, we present endocytome profiling, a proximity-labeling approach that captures the dynamic protein exchange between the plasma membrane and endosomes within genetically defined cell types in the brain. We applied this method to profile a sub-population of axons during development, a stage that requires reorganization of CSP-mediated adhesion and signaling. Quantitative proteomic analysis revealed that parallel profiling of the surface and endosomes is essential to determine each protein’s primary residence and yielded a compartment-resolved proteomic map comprising endolysosomal residents, CSPs, and secreted proteins. We leveraged these data to uncover a novel mechanism by which endocytosis remodels the CSP landscape to control the refinement and long-term stability of neural circuits. We also demonstrate that this dataset can be used to identify transcellular signaling events that shape circuit connectivity. Endocytome profiling thus provides a powerful tool for decoding proteome-scale dynamics across cell states and systems.

## RESULTS

### Endocytosis is critical for ORN target selection

**Figure 1.**
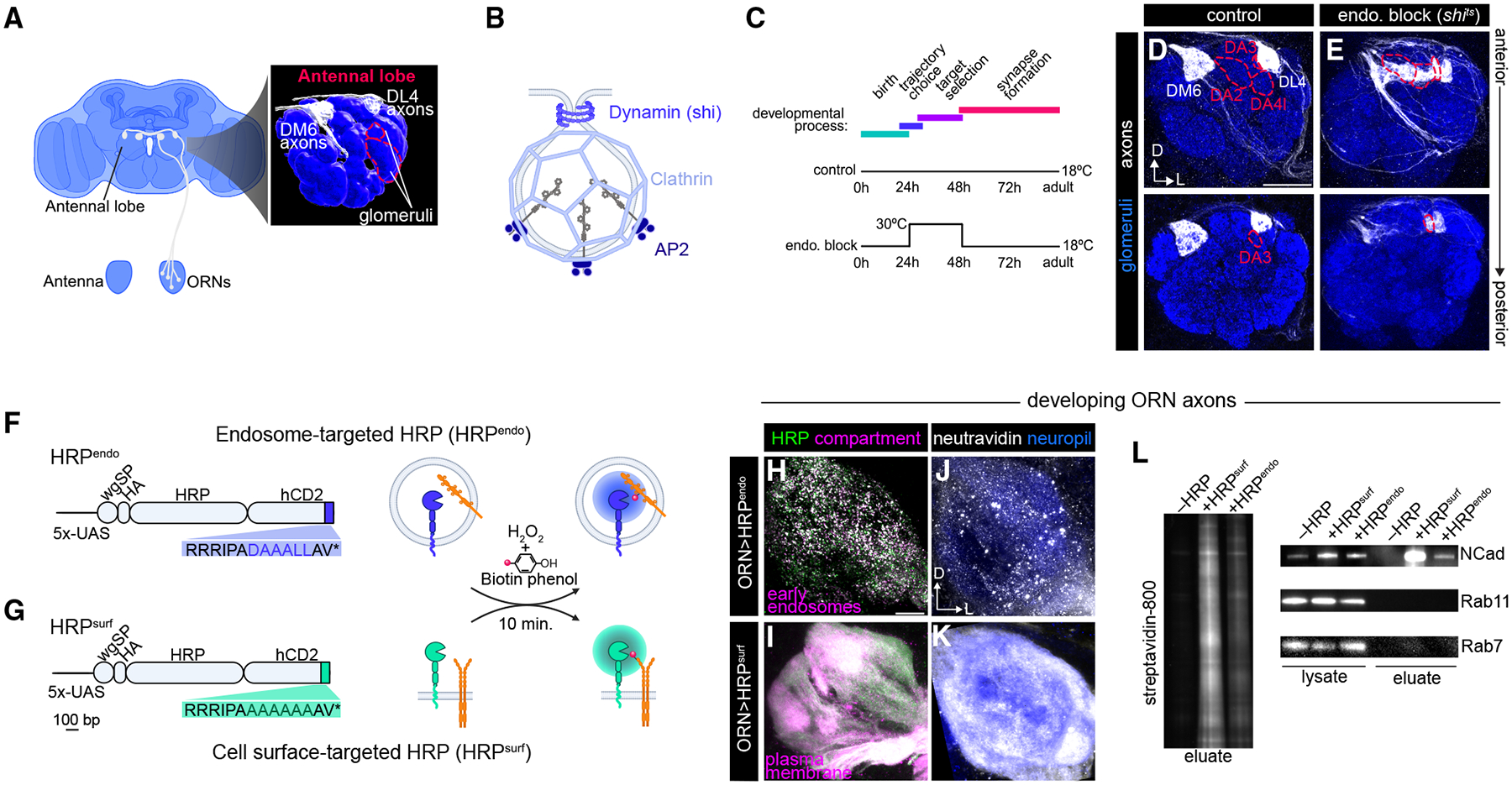
Endocytic control of ORN axon targeting and development of endocytome profiling tools (A) Adult *Drosophila* brain schematic highlighting the olfactory system (left) and the antennal lobe depicting the axon trajectories of two ORN subtypes and their terminals in the DM6 and DL4 glomeruli (right). (B) Endocytic vesicle and the machinery necessary for clathrin-mediated endocytosis. (C) ORN development (top) and the temperature shift paradigm used in controls (middle) or to inhibit dynamin function during axon target selection (bottom). Hours after puparium formation (APF) (h) are based on 25°C. (D and E) Images of ORN axons innervating the DM6 and DL4 glomeruli (white, labeled with membrane-targeted tdTomato) that express *UAS-shi*^*ts*^ via the *AM29-GAL4* driver of controls (D) and experimental flies (E). Dotted outlines denote glomeruli in which ectopic targeting is observed. Scale bar, 20 μm. (F and G) Endosome-targeted (F) and plasma membrane-targeted (G) HRP transgenes (left) and labeling paradigm (right). wgSP, wingless signal peptide; bp, base pair. (H–K) Airyscan super-resolution images of 36 h APF antennal lobes in which all ORNs express either *UAS-HA-HRP*^*endo*^ colocalized with early endosome marker *UAS-mCherry-2xFYVE* (H) or *UAS-HA-HRP*^*surf*^ colocalized with *UAS-myristoylated-RFP* (I). NeutrAvidin staining when *HRP*^*endo*^ (J) or *HRP*^*surf*^ (K) was expressed in ORN axons. Scale bar, 10 μm. (L) Post-enrichment bead eluate probed with streptavidin (left) and immunoblots of lysate and post-enrichment eluate stained for NCad and Rab proteins (right). Here and in all subsequent figures: D, dorsal; L, lateral. NCad (in blue) is used to label neuropil/glomeruli. *p < 0.05; **p < 0.01; ***p < 0.001; ****p < 0.0001; ns, not significant. *n* indicates the number of the antennal lobes quantified. Images were from antennal lobes of young adults (<5 days old), unless otherwise noted. Detailed information regarding genotypes can be found in Table S1. See Figures S1 and S2 for additional data.

CSP endocytosis is known to influence axon guidance^15,16^; however, its role in the subsequent step of target selection—in which neurons choose specific postsynaptic partners from many—remains unclear. We investigated this using *Drosophila* olfactory receptor neurons (ORNs), the primary sensory neurons of the olfactory system. Upon entering the brain, axons of the ~50 distinct ORN subtypes follow a specific trajectory and target the dendrites of their postsynaptic partners, one of ~50 subtypes of projection neurons (PNs), to form one-to-one synaptic connections within discrete, anatomically stereotyped units called glomeruli (Figure 1A).

Clathrin-mediated endocytosis is the primary receptor internalization pathway in neurons. It involves the sequential recruitment of adaptor proteins, such as AP2 to cluster endocytic cargos, followed by clathrin, and GTPase dynamin, which mediates vesicle scission from the plasma membrane^18,19^ (Figure 1B). Since earlier axon guidance decisions, such as trajectory choice, influence target selection,^20^ we temporally restricted endocytic inhibition to the latter process by expressing a temperature-sensitive *shibire* transgene (*shi*^*ts*^, encoding a dominant-negative form of dynamin) only in ORN axons projecting to the DM6 and DL4 glomeruli (Figures 1A and 1D). *shi*^*ts*^ only blocks dynamin function when flies are at high temperatures (29°C–31°C),^21^ giving us precise control over when endocytosis is disrupted.

Rearing these flies at 30°C for a 24-h window after the majority of axons have selected their trajectory^22^ but before target selection and synapse assembly^23^ resulted in robust and specific defects in ORN targeting (Figures 1C–1E). DM6-ORN axons exhibited a significant reduction in targeting to their normal glomerulus and a significant increase in innervation to two specific glomeruli, DA2 and DA4l, when dynamin activity was blocked (Figures 1E and S1A). Similarly, impairing the expression or function of AP2 or clathrin led to stereotyped mistargeting of DM6-ORN axons (Figures S1C and S1D). Likewise, blocking dynamin activity in DL4-ORNs caused them to mistarget to the DA3 glomerulus (Figures 1E and S1B). The fact that impairing endocytosis during a specific developmental window caused ORN axons to innervate stereotyped but incorrect glomeruli suggests that this process regulates a specific repertoire of CSPs necessary for axon target selection.

### Proximity-labeling tools for profiling endocytic and cell-surface proteomes

To comprehensively define the CSPs enriched in endosomes during axon targeting, we developed a quantitative proximity-labeling approach to chart the surface-to-endosome distribution of CSPs in developing ORN axons. CSP selection for clathrin-mediated endocytosis can occur via short amino acid motifs in their cytoplasmic tails.^24,25^ Thus, we targeted the proximity-labeling enzyme horseradish peroxidase (HRP) to the lumen of endosomes (hereafter HRP^endo^) by placing it N-terminal to the extracellular domain of human CD2 (a single-pass transmembrane [TM] protein) and adding a dileucine-based endocytic motif to its cytoplasmic tail (Figure 1F). The entire open reading frame is under *UAS* control for GAL4-mediated cell-type-specific expression (Figure 1F). We validated that HRP^endo^ was internalized *in vitro* and *in vivo* (Figures S2A and S2C) and that it colocalized with markers of the endolysosomal system (Figures S2B and S2D–S2F). Further, in developing ORN axons, HRP^endo^ partially colocalized with mCherry-2xFYVE, which predominantly accumulates on early endosomes^26^ (Figure 1H), validating that it was in the correct subcellular compartment in our cells of interest. Together, these data indicate that clathrin-mediated endocytosis provides an efficient route to deliver HRP into endosomes.

We labeled CSPs residing on the plasma membrane by mutating the dileucine motif of HRP^endo^ to alanines, hereafter referred to as HRP^surf^ (Figure 1G). HRP^surf^ should take the same secretory route as HRP^endo^ to the plasma membrane but should not robustly enter endosomes. We validated that HRP^surf^ was predominantly at the plasma membrane in ORN axons and cultured cells (Figures 1I, S2A, and S2B).

Upon H_2_O_2_ application, both HRP^endo^ and HRP^surf^ labeled proteins using the membrane-permeable biotin-phenol in dissected brains (Figures 1J and 1K). The biotin distribution reflected their respective subcellular compartments, with HRP^endo^ labeling appearing punctate and HRP^surf^ being continuous throughout ORN axons (Figures 1J and 1K). Streptavidin bead enrichment confirmed labeling of a wide range of proteins (Figure 1L, left), and immunoblots validated compartment specificity—detecting the neuronal surface protein N-Cadherin (NCad), but not cytosolic endosome-associated Rab GTPases Rab7 and Rab11 in streptavidin bead eluates (Figure 1L, right). These results are consistent with HRP’s orientation toward the extracellular and luminal space and the membrane impermeability of the biotin-phenoxyl radical it generates, which prevents labeling of cytosol-residing proteins, such as Rabs.

### The axonal endocytome and surfaceome are enriched for distinct proteins

**Figure 2.**
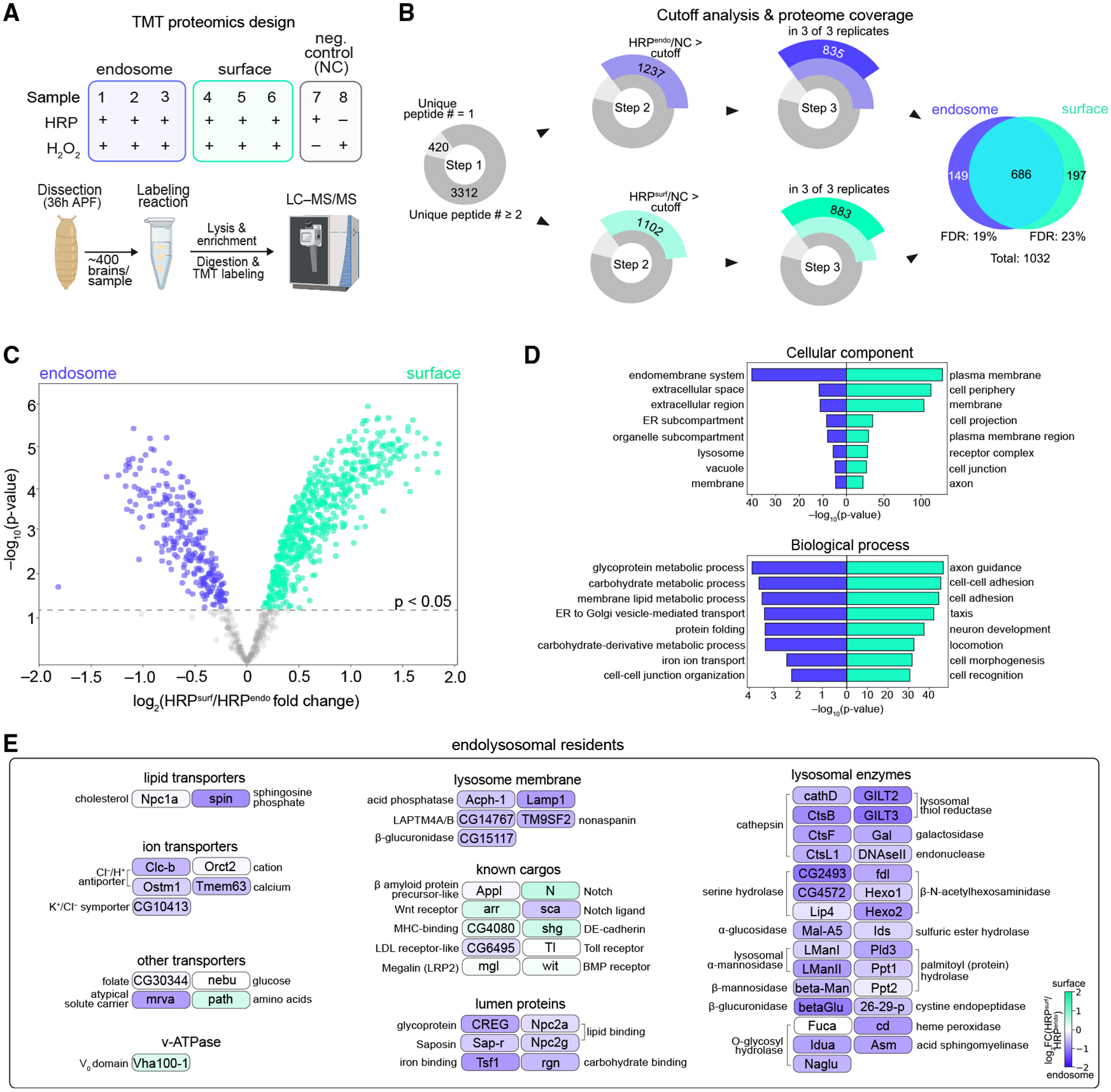
Proximity labeling enables profiling of global CSP dynamics in ORN axons (A) Design of the tandem mass tag (TMT)-based quantitative proteomics experiment. Each genotype comprises three biological replicates (blue or green) in addition to two negative controls (gray). (B) Summary of cutoff analysis applied to the proteomes and number of proteins retained after each filter. FDR, false discovery rate. (C) Volcano plot depicting individual protein enrichment in the axonal endocytome or surfaceomes. Dashed line denotes *p* value cutoff applied to proteomes yielding 571 surfaceome and 248 endocytome proteins used for GO analysis. A moderated *p* value is depicted as a cutoff. (D) Top 8 GO categories: cellular component (top) and biological process (bottom). (E) Schematic of proteins known to be in the endolysosomal system. Color indicates level of endosome or surface enrichment (in log_2_[HRP^surf^/HRP^endo^] fold change). The surfaceome was enriched for two known endolysosomal residents, including a V_0_ vacuolar H^+^-ATPase subunit and an amino acid transporter (path), both with known endolysosomal and plasma membrane localization.^28,29^ See Figure S3 and Table S2 for additional data.

Next, we combined HRP-mediated biotinylation with mass-spectrometry-based proteomics to define the “endocytome” and “surfaceome” of ORN axons. We specifically profiled ORN axons during target selection, as we could easily dissect distal axons away from their cell bodies in the antenna (Figure 1A). Using a tandem mass tag (TMT)-based quantitative strategy,^27^ we profiled three biological replicates of HRP^endo^ or HRP^surf^ alongside two negative controls (lacking either HRP or H_2_O_2_) to account for non-specific labeling (Figure 2A).

From the 3,732 total proteins detected by mass spectrometry, we identified compartment-enriched proteins via four filtering steps. First, we retained the 3,312 proteins with at least two unique peptides detected (Figure 2B; Table S2). Second, we ranked proteins by their experimental-to-negative-control TMT ratios in descending order and validated that proteins containing a signal peptide and/or a TM domain were enriched, whereas putative contaminants lacking these signatures were not (Figures S3A–S3D). Following published protocols,^6,30^ we filtered out contaminants using this ratiometric approach (Figure 2B). Third, since HRP^endo^ or HRP^surf^ replicates were highly similar (Figure S3E), we retained proteins found in all three proteomes per condition (Figure 2B). These steps yielded a total of 1,032 proteins, most of which were detected by both HRP^endo^ and HRP^surf^ labeling (Figure 2B), highlighting the dynamic nature of protein localization. Finally, to determine compartment-specific enrichment, we compared the extent of biotinylation of these proteins using their relative abundance in HRP^endo^- vs. HRP^surf^-labeled proteomes. This yielded 248 and 571 proteins that were significantly enriched in the endocytome and surfaceome, respectively (Figure 2C).

Confirming the spatial specificity of our approach, Gene Ontology (GO) analysis classified endocytome proteins within the extracellular region, endomembrane systems, and lysosomes, whereas surfaceome proteins localized to the plasma membrane and cell periphery (Figure 2D). Consistent with the stage profiled, the top biological process GO terms in the surfaceome were related to axon development and cell adhesion (Figure 2D). Notably, cell-cell junction organization terms were highly enriched in the endocytome (Figure 2D), implying that internalization of this specific class of proteins is important for axon development (see Figures 4 and 5). The endocytome was enriched for metabolic and ion-transport terms (Figure 2D), consistent with its role in macromolecule degradation. A deeper investigation of the proteins in these categories revealed lysosomal enzymes, integral membrane proteins, and membrane transporters (Figure 2E), establishing a comprehensive atlas of endolysosomal residents and further validating our profiling.

Our endosomal profiling also captured crosstalk between the secretory pathway and endolysosomal network as evidenced by the presence of endoplasmic reticulum (ER)-related GO terms (Figure 2D). Protein exchange between these two systems is well documented^31,32^ and proteins that enter the endolysosomal system via biosynthetic vesicles typically participate in degradative processes^33,34^ or are themselves targeted for degradation.^35^ In fact, several of these proteins have been implicated in a recently identified ER-to-lysosome degradation pathway.^36–40^ These findings highlight that endocytome profiling detects proteins entering the endolysosomal system from both the plasma membrane and biosynthetic compartments.

### Dual-compartment profiling is required to resolve protein localization

Prior studies have used cell-surface proximity labeling to define changes in the steady-state protein composition across development^6,7^ and identified internalized CSPs by profiling immunoprecipitated neuronal endosomes.^41^ Although useful for characterizing compartment-specific proteomes, we reasoned that profiling each compartment in isolation would not only provide less quantitative insight into a protein’s distribution across compartments but could skew datasets toward highly abundant proteins rather than those enriched in each one.

**Figure 3.**
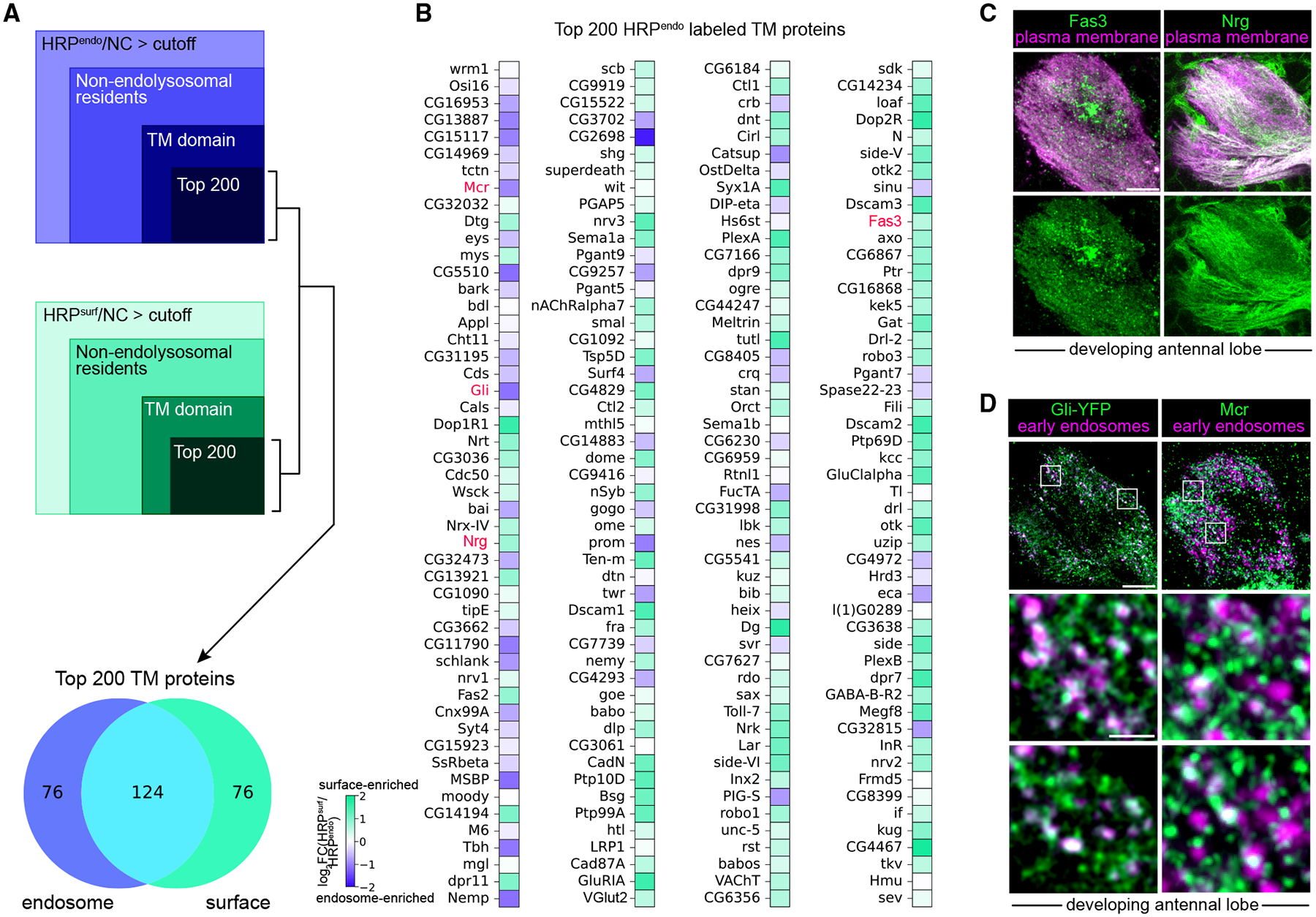
Dual-compartment profiling is required to resolve preferential protein localization (A) Filters used to identify the top 200 transmembrane (TM) proteins labeled by either HRP^endo^ (top) or HRP^surf^ (middle) and a Venn diagram (bottom) of the proportion of the TM proteins that are labeled by either/both HRP transgenes. (B) Heatmaps depicting endosome and surface enrichment (in log_2_[HRP^surf^/HRP^endo^] fold change) of the top 200 TM proteins labeled by HRP^endo^. Proteins are rank-ordered from highest to lowest, labeled by HRP^endo^. Enrichment of TM proteins in pink was validated in (C) and (D). Note: some of the top proteins regulate secretory pathway-to-endolysosomal network trafficking or degradation (detailed in Figure 2). (C) Colocalization of myristoylated-RFP expressed only in ORNs and surface-enriched proteins Fas3 (left) and Nrg (right). Note that based on anatomy and membrane staining, the puncta in Fas3 images are largely from PNs. Images were taken at ~36 h APF. Scale bar, 5 μm. (D) Airyscan super-resolution images of all ORN axons expressing the early endosome marker mCherry-FYVE and endogenously tagged Gliotactin (Gli)-YFP (left) and anti-Mcr (right). Images were taken at ~36 h APF. Scale bar, 10 μm (D) and 1 μm (D, zoom). See Table S3 for further information.

We directly tested the extent to which protein abundance impacts labeling by examining the top 200 TM proteins labeled by HRP^endo^ before comparing them with those labeled by HRP^surf^ (Figure 3A). Indeed, 62% of these proteins were also among the top 200 TM proteins labeled by HRP^surf^ (Figure 3A). This overlap led us to ask how these proteins were distributed between the surface and endosomes. In fact, many were more enriched at the cell surface (Figure 3B), likely reflecting high overall protein levels. To validate these findings, we analyzed the localization of four representative proteins in ORN axons and found that their spatial distribution mirrored the compartment assignments from our ratiometric analysis (Figures 3C and 3D). Many of the top 200 TM proteins labeled by HRP^endo^ had human orthologs and were expressed in human cortical neurons (Table S3). These data emphasize that determining a TM protein’s primary residence requires concurrent profiling of both compartments.

Collectively, these data establish proximity labeling as a powerful strategy for identifying proteins regulated by the endolysosomal network within a subset of axons in the intact brain. To showcase the utility of this tool in revealing how endocytic remodeling equips neurons to meet distinct developmental demands, we pursued two complementary approaches: (1) defining how the spatial distribution of CSPs instructs their function and (2) leveraging multi-omics to uncover cell-cell interactions underlying circuit assembly.

### The endocytome contains multiple cell-cell junction proteins required for axon development

We first focused on the CSP cargos most enriched in endosomes. In contrast to the surfaceome in which many canonical neurodevelopment proteins were detected (Figures S4A and S4B), cell-cell junction CSPs were highly enriched in the endocytome (Figure 2D). These junctions include septate, tight, and adherens junctions and are formed by CSPs that link cells to one another or to the extracellular matrix.^42^ In the nervous system, such junctions form between progenitors, glia, and myelin and axons,^43–45^ but not between neurons. Since ORN axons have minimal glial contact during target selection,^46,47^ the endosomal enrichment of these junctional proteins was unexpected.

**Figure 4.**
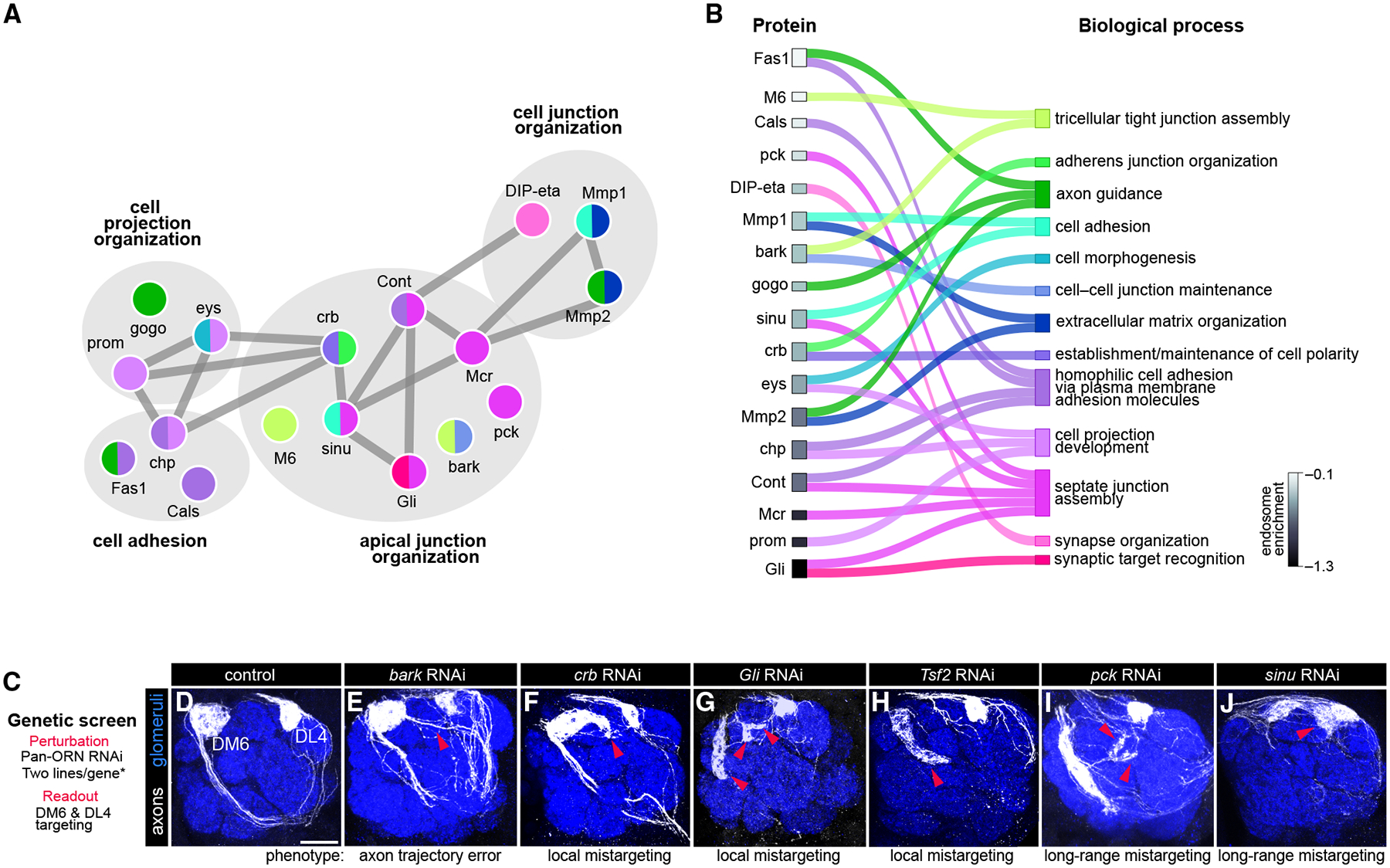
The endocytome contains multiple cell-cell junction proteins critical for axon targeting (A) STRING plot depicting cell-cell junction proteins and the other endosome-enriched CSPs they interact with. Links between nodes denote protein-protein interactions, and node colors depict the category assignment(s) in (B). (B) Chord plot depicting the specific process(es) CSPs from (A) are associated with. Proteins are rank-ordered from lowest to highest endosomal enrichment (top to bottom). Gray scale in rectangles indicates level of enrichment (log_2_[HRP^surf^/HRP^endo^] fold change). (C) Details of the loss-of-function genetic screen in which endosome-enriched CSPs were knocked down in all ORNs, and targeting of axons projecting to the DM6 and DL4 glomeruli were monitored. Asterisk denotes that only one RNAi line was used for *Tsf2* knockdown as all others caused lethality. (D–J) Images of antennal lobes of indicated genotypes. Phenotypes and phenotypic penetrance are detailed in Table S4. Arrowheads indicate DM6-ORN axon mistargeting. Scale bar, 20 μm. See Figure S4 and Table S4 for additional data.

This prompted us to perform focused analysis of proteins in the cell-cell junction, cell adhesion, and cell projection organization categories, as many of them interact (Figure 4A). We annotated each protein with the specific biological process(es) it was most implicated in. A few proteins had known neuronal roles, but many did not (Figure 4B). Our prior colocalization experiments confirmed endosomal enrichment of two junctional proteins, Macroglobulin complement-related (Mcr) and Gli (Figure 3D).

Next, we conducted a targeted loss-of-function screen to evaluate whether these junctional proteins have roles in axon development. We expressed RNAi lines against each gene in all ORNs and monitored axons projecting to the DM6 glomerulus (Figure 4C). All six genes tested produced axonal phenotypes (Figures 4D–4J; Table S4). For example, the scavenger receptor bark beetle (bark) promoted axon trajectory choice but did not strongly affect later developmental events (Figure 4E). Loss of the adherens junction protein *crumbs* (*crb*) or septate junction components *Transferrin 2* (*Tsf2*) and *Gli* caused axons to mistarget to glomeruli nearby DM6 (local mistargeting; Figures 4F–4H), whereas knockdown of the claudins *pickel* (*pck*) and *sinuous* (*sinu*) caused long-range mistargeting (Figures 4I and 4J). These data indicate that junctional CSPs control distinct aspects of axon targeting. A recent study found transcripts for tight and adherens junction CSPs enriched in mammalian neurons during neurite outgrowth and synaptogenesis,^4^ suggesting that this class of proteins may act as conserved regulators of circuit assembly.

Since this is the first profiling of the ORN surfaceome, we also examined whether surface-enriched CSPs regulate axon development. 5 of the 6 genes we tested controlled one or more aspects of axon targeting (Figures S4C–S4I; Table S4). The fact that 91% (10/11) of the CSPs tested produced phenotypes validates both the biological relevance of our profiling and the idea that circuit assembly depends on multiple CSPs distributed between endosomes and the plasma membrane.

### Crumbs promotes axon pruning and is endocytosed to safeguard axon integrity

**Figure 5.**
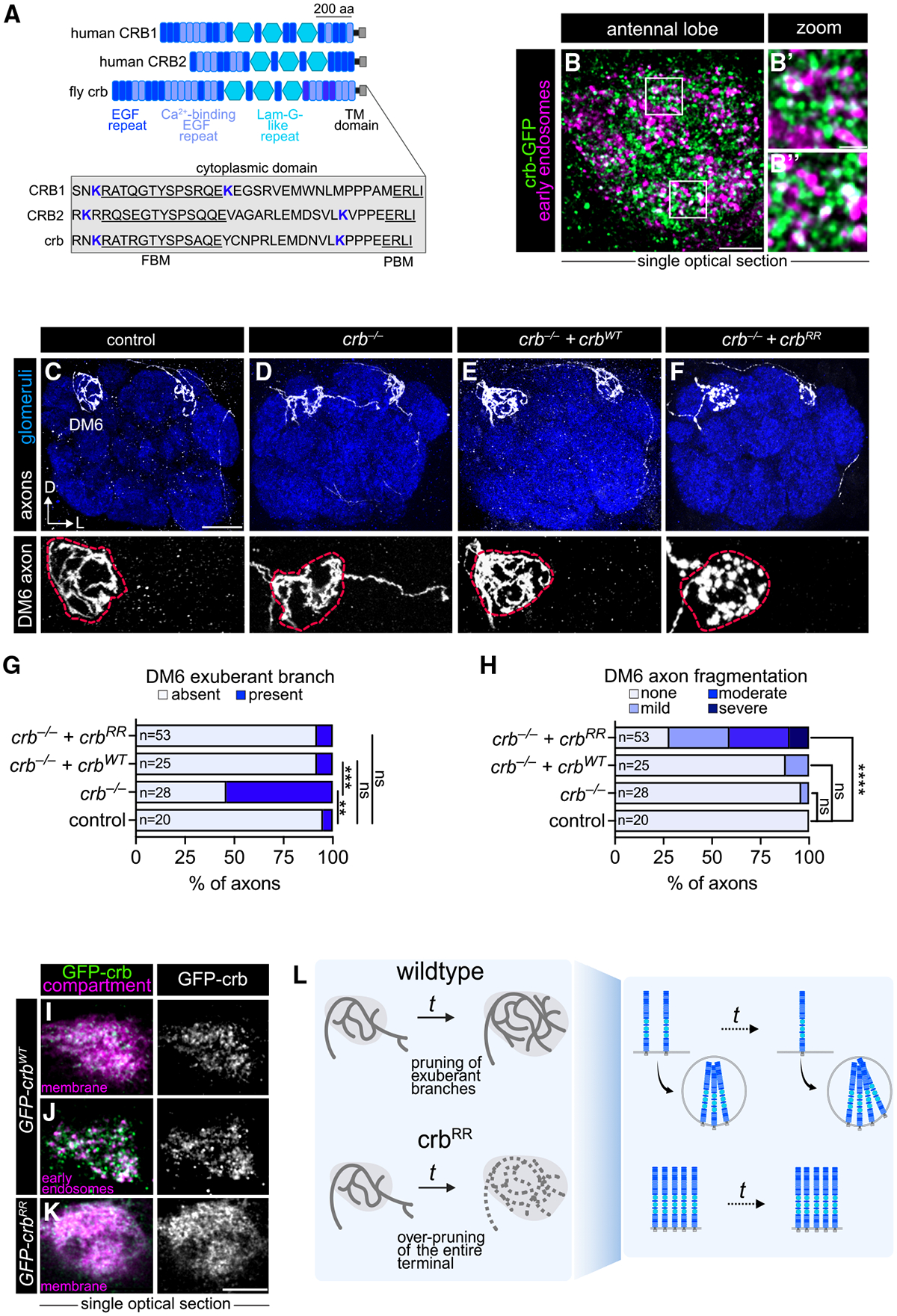
Crumbs promotes axon pruning and is endocytosed to safeguard axon integrity (A) Top: domain structure (based on UniProt) of human and *Drosophila* crumbs (crb) proteins. Bottom: the entire cytoplasmic domain is highlighted in the gray box with the two lysines (K) that facilitate fly crb endocytosis in blue. FBM, FERM-domain binding motif; PBM, PDZ-domain binding motif; aa, amino acids. (B) Airyscan super-resolution image of all ORN axons expressing early endosome marker mCherry-FYVE and endogenously tagged crb-GFP allele. Zoomed-in images of (B′ and B″) show crb-GFP colocalization with mCherry-FYVE. Images were taken at ~36 h APF. (C–F) Images of single DM6 and DL4 axons of indicated genotypes from *hs-FLP*-based MARCM clones (top) and zoomed-in images of the DM6 axons depicting membrane integrity (bottom). Dashed lines denote the DM6 glomerular boundary. (G and H) Quantification of the percentage of DM6 axons that still have the exuberant branch present at the adult stage (G) and axon fragmentation (H) of the indicated genotypes. (I–L) Airyscan super-resolution images of 40–48 h APF axons overexpressing *UAS-GFP-Crb*^*WT*^ or *UAS-GFP-Crb*^*RR*^ colocalized with *mtdTomato* or *mCherry-2xFYVE*. (M) Summary of crb function in ORN axons. Fisher’s exact test was used to determine statistical significance. Scale bar, 10 μm (B), 1 μm (B′ and B″), 20 μm (C), and 5 μm (L). See Figure S5 for additional data.

How does endocytosis shape the function of junctional CSPs? We addressed this question by studying crb, an evolutionarily conserved protein involved in epithelial polarity,^48,49^ adherens junction formation,^50^ and photoreceptor integrity.^51,52^ Humans have three CRB proteins, with CRB1 and CRB2 being most similar to the single fly crb (Figure 5A, top). Though CRB1/2 are expressed in the brain,^53,54^ the neuronal role(s) of any crb protein in this tissue is largely undefined.

According to our developmental single-cell RNA sequencing (scRNA-seq) data,^3,5^ c*rb* is expressed in ORNs, but not their postsynaptic partner PNs (Figure S5A), indicating that crb in our endocytome is likely produced autonomously by ORNs. Using an endogenously tagged *crb* allele^55^ (*crb-GFP*), we found that crb-GFP proteins appeared punctate and partially colocalized with early endosomes in developing ORN axons (Figures 5B′ and 5B″), with non-colocalized puncta likely falling within other endosomal compartments.

Having validated our proteomic identification of crb as an endosome-enriched protein, we next investigated its function in axon development. Using mosaic analysis with a repressible cell marker (MARCM),^56^ we probed the cell-autonomous role of crb using a null allele.^57^ We used either *eyeless-FLP* (*eyFLP*) or *heat shock-FLP* (*hs-FLP*) to induce large (in 30%–50% of ORNs) or small (down to single ORN) MARCM clones, respectively, wherein only homozygous *crb* mutant axons expressed a membrane marker in DM6- and DL4-ORN axons. Mutant clones displayed significantly increased exuberant branches extending beyond the DM6 glomerulus compared with controls (Figures 5C, 5D, 5G, S5B, S5C, and S5F). This phenotype was rescued by expressing wild-type crb (*UAS-GFP-crb*^*WT*^) only in labeled ORNs (Figures 5E, 5G, S5D, and S5F), validating that crb acts cell autonomously. During development, DM6 axons send branches to neighboring glomeruli that are later pruned to form the adult circuit^22^ (Figures S5H–S5J). Their persistence in *crb* mutants suggests that crb promotes the pruning of exuberant branches.

Does endocytosis play a role in crb-dependent pruning? Crb’s highly conserved 37-amino-acid cytoplasmic tail contains two lysine (K) residues (Figure 5A) whose ubiquitination drives endocytosis.^58^ We compared the distribution of crb proteins produced from the wild-type crb rescue transgene and an endocytosis-deficient version (*UAS-GFP-crb*^*RR*^), which is identical in sequence, genomic insertion site, and protein expression levels (Figure S5M) except that two cytoplasmic lysines (K) are mutated to arginines (R). Crb^WT^ was punctate and partially colocalized with an early endosome marker (Figures 5I and 5J). Crb^RR^ was uniformly distributed across the membrane (Figure 5K) and was largely absent from the early endosomal compartment (Figure 5L), consistent with impaired internalization.

Expression of *crb*^*RR*^ in *crb* mutant ORNs did not alter the number of exuberant branches present in adult DM6 axons (Figures 5F, 5G, S5E, and S5F), indicating that crb endocytosis is not necessary for axon pruning. Furthermore, developing crb^RR^ axons extended a similar proportion of branches to nearby glomeruli as controls (Figures S5K and S5L), demonstrating that restricting crb to the plasma membrane does not impair normal features of axon development. Collectively, these results demonstrate that crb at the plasma membrane, not the endosome, is necessary for exuberant branch removal.

Since our data imply that crb promotes developmental axon pruning, we asked whether sustained surface crb localization causes over-pruning of mature axons. Strikingly, inhibiting crb endocytosis caused axons to become fragmented (Figures 5F, bottom, 5H, S5E, and S5G)—hallmarks of axon instability. There were no changes in the number of ORN cell bodies in *crb*^*RR*^ compared with controls (Figure S5N), suggesting that this phenotype is not a consequence of cell death. Thus, prolonged surface localization of crb leads to over-pruning of the entire axon terminal. We propose that the precise balance of crb between endosomes and the cell surface enables axon pruning during development while preserving terminal integrity in the mature circuit (Figure 5M). Altogether, these findings highlight how spatially resolved CSP profiling can uncover critical mechanisms that coordinate developmental circuit refinement and long-term circuit stability.

### Multi-omics reveals ligand-receptor pairs internalized during axon targeting

**Figure 6.**
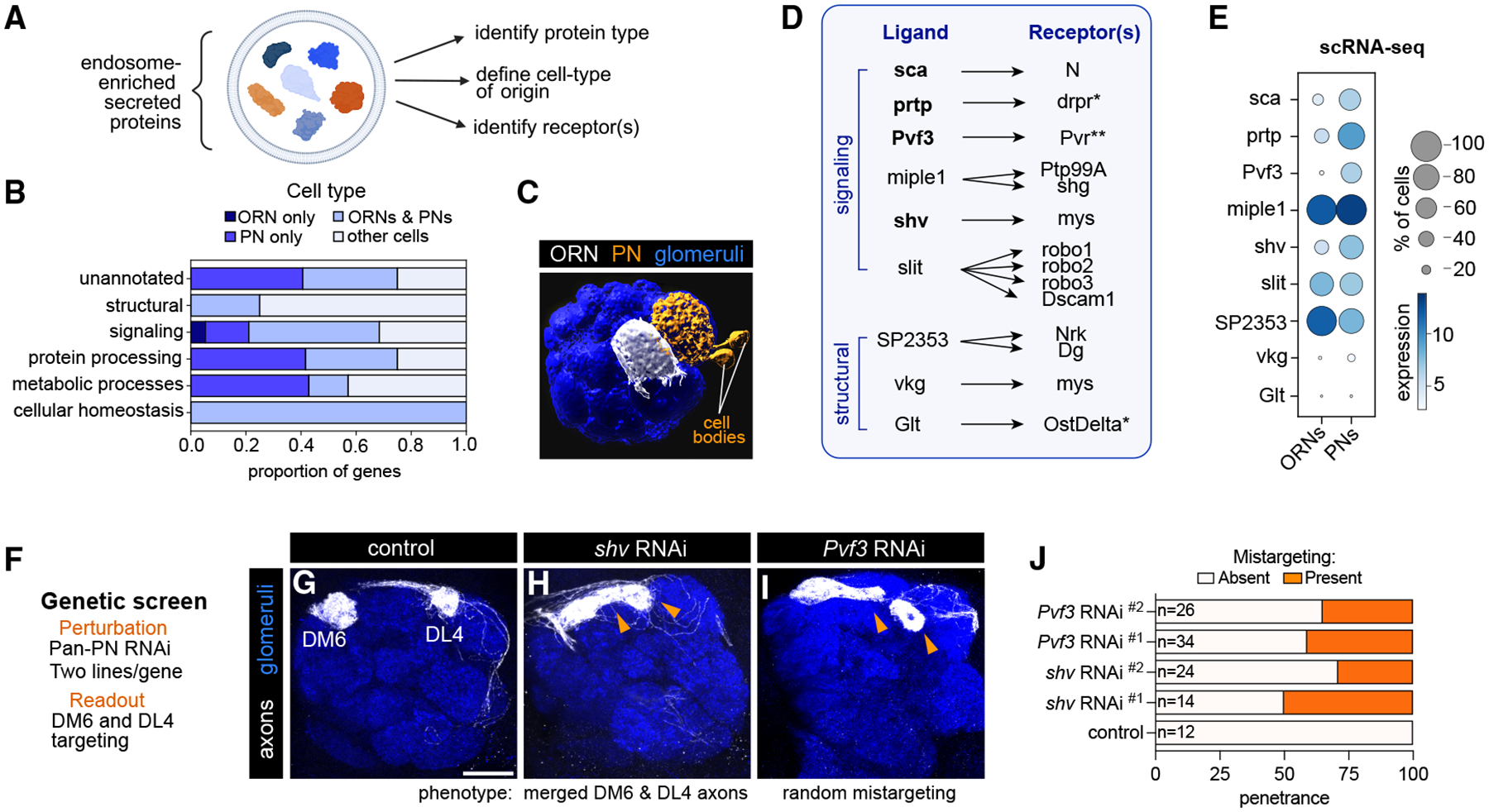
Multi-omic analysis reveals ligand-receptor pairs internalized during axon targeting (A) Workflow for identifying endocytosed ligand-receptor pairs. (B) Stacked bar plot depicting the cell type(s) in which secreted proteins are robustly expressed (log_2_[CPM+1] ≥ 4 in ≥30% of cells). Transcripts not expressed in ORNs or PNs are likely from glia or local interneurons whose transcriptomes have not been profiled. (C) Volume rendering of the fly antennal lobe, which contains the axons of ~50 distinct subtypes of ORNs and the dendrites of their ~50 distinct subtypes of PNs forming one-to-one connections at ~50 glomeruli. Projections of one ORN type (white) and one PN type (orange) that innervate adjacent glomeruli are shown. (D) Potential ligand-receptor pairs found in ORN endosomes. Bolded ligands are more abundantly expressed in PNs. *, endosome-enriched; **, similarly labeled in endocytome and surfaceome. (E) Dot plot depicting expression levels of transcripts encoding ligands in (D) in ORNs or PNs. scRNA-seq expression is log_2_(CPM+1). CPM, counts per million reads. Dot plot depicts averaged expression in all PN or ORN types at 24 and 48 h APF. (F) Details of the loss-of-function genetic screen in which secreted proteins were knocked down in all PNs, and axons projecting to the DM6 and DL4 glomeruli were monitored. (G–I) Images of antennal lobes of indicated genotypes. (J) Quantification of phenotypic penetrance for DM6 axons in indicated genotypes. Scale bar, 20 μm. See Figures S6 and S7 for additional data.

The endocytome also contained a diversity of secreted proteins. Because these proteins can originate from neighboring neurons or glia and enter ORNs via receptor-mediated internalization, we asked whether this dataset could be further leveraged to infer cell-cell interactions (Figure 6A).

The 84 endosome-enriched secreted proteins (Table S2) spanned five functional categories: (1) cellular homeostasis, (2) protein processing, (3) signaling, (4) metabolic processes, and (5) structural roles (Figure 6B), highlighting the rich extracellular milieu axons are navigating. Signaling proteins were the largest category, containing both classic neurodevelopment regulators (e.g., slit [sli], and scabrous [sca]) and proteins not previously linked to development (Table S2). To begin decoding cell-cell communication underlying axon development, we asked which cell types secreted the proteins internalized by ORNs. Using our developmental scRNA-seq data,^3,5^ we mapped the expression of each protein to ORNs or PNs (Figure 6C). Many transcripts were shared between ORNs and PNs (Figures 6B and S6). Although only two transcripts were abundantly expressed in ORNs, over 35% of transcripts in the unannotated, protein processing, and metabolic process categories, as well as 15% of signaling transcripts, were more abundant in PNs (Figure 6B). To corroborate these cell-type assignments, we experimentally validated PN-specific expression of two such transcripts, *Pxn* and *sca* (Figure S7). Together, these data indicate that endocytome profiling is sensitive enough to detect cues secreted by neighboring cells and endocytosed by ORN axons.

We next evaluated whether any of the known receptors for these secreted proteins were present in endosomes. Indeed, receptors for several signaling and structural proteins were endocytosed (Figure 6D). Although not all receptors were endosome enriched (Figure 6D), these data nevertheless support ligand entry via receptor binding and internalization. Lastly, we tested whether any of the ligands are secreted by PNs to regulate ORN axon targeting (Figure 6F). We focused on *Pvf3* and *shv*, as both were more abundant in PNs (Figure 6E) and neither has been linked to neuronal development. Knockdown of *shv* (encoding a soluble ß-integrin [mys] ligand) in all PNs caused DM6-ORNs to mistarget laterally and at times fuse with DL4 axons (Figures 6H and 6J), whereas knockdown of *Pvf3* (see below) led to random mistargeting of DM6 axons and widespread glomerular disorganization, including fusion of some glomeruli and loss of others, consistent with a global disruption of circuit assembly (Figures 6I and 6J). Altogether, these analyses highlight how cell-type-specific endosome profiling reveals transcellular signaling critical for axon targeting.

### Endocytosis tunes Pvr levels to match target-derived Pvf3 for axon targeting

**Figure 7.**
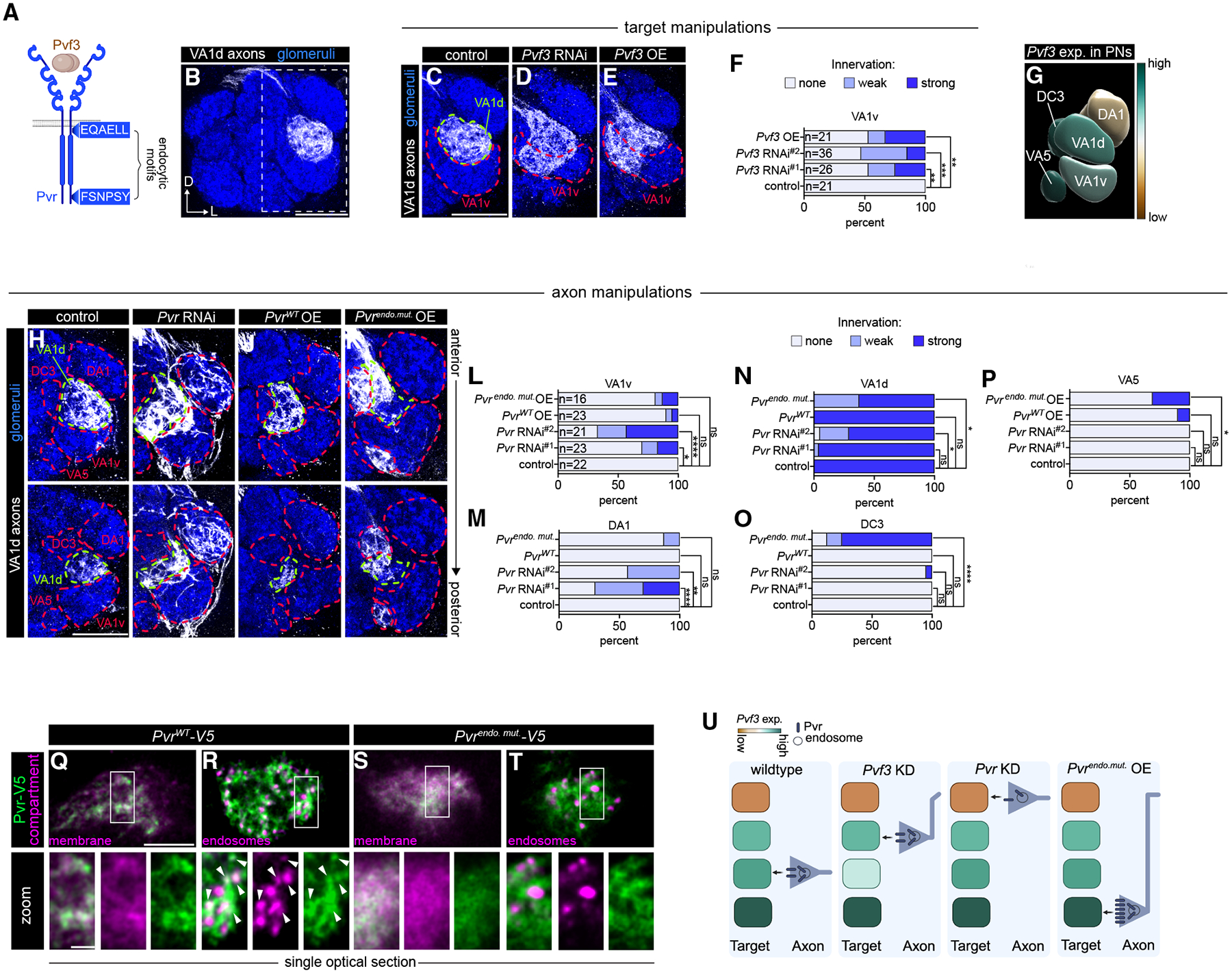
Endocytosis tunes Pvr levels to match target-derived Pvf3 for axon targeting (A) Schematic of Pvf3 and Pvr. Boxes indicate endocytic motifs and their approximate location flanking the tyrosine kinase domain. (B) Image of the antennal lobe depicting VA1d-ORN axons. The dashed rectangle indicates glomeruli that are depicted in subsequent panels. (C–E) Images of antennal lobes depicting targeting of VA1d-ORN axons when *Pvf3* expression is manipulated in PNs. (F) Quantification of VA1d-ORN axon targeting. (G) Volume rendering depicting *Pvf3* expression in glomeruli as analyzed in the prior and subsequent panels. Data used to generate the expression heatmap overlay are shown in Figures S8B and S8C. Expression is in arbitrary units. (H–K) Images of the antennal lobe depicting targeting of VA1d-ORN axons when *Pvr* is manipulated. (L–P) Quantification of VA1d axon targeting. *n*s for all panels are in (L). (Q–T) Airyscan super-resolution images of 40–48 h APF axons overexpressing *UAS-Pvr*^*WT*^*-V5* or *UAS-Pvr*^*endo.mut*.^*-V5*, along with *mtdTomato or mCherry-2xFYVE*. Arrowheads denote colocalized puncta. (U) Summary of Pvf3-Pvr function in axon targeting. Fisher’s exact test was used to determine statistical significance. Scale bars, 20 μm (B, C, and H), 5 μm (Q, top), and 2 μm (Q, bottom). See Figures S8–S10 for additional data.

Finally, we investigated how the internalization of one of the ligand-receptor pairs we discovered, Pvf3-Pvr, contributes to axon target selection. Pvf3 is one of three fly orthologs of both platelet-derived growth factor (PDGF) and vascular endothelial growth factor (VEGF)^59,60^ and the only one expressed in PNs (Figure S8A). Pvr is a receptor tyrosine kinase (Figure 7A) and the sole fly ortholog of the PDGF and VEGF receptors.^59^ This pair has been shown to regulate phagocyte migration,^61^ but their role in neural development is undefined.

We first sought to comprehensively characterize Pvf3’s expression in PNs to better understand how its loss caused such widespread disruptions in antennal lobe morphology (Figure 6I). Analysis of scRNA-seq revealed differential expression of *Pvf3* between PN types (Figure S8A); however, not all PN types are present in this dataset. To validate and extend this analysis, we used an intersectional *GAL4*-based reporter strategy that largely mirrored the *Pvf3* expression pattern observed in our PN transcriptomes (Figures S8B and S8C). The marked heterogeneity in *Pvf3* expression across PN types led us to hypothesize that cell-type-specific ligand levels instruct ORN axon targeting.

Having genetic access to both VA1d-PNs and VA1d-ORNs during development enabled us to investigate the role of PN-derived Pvf3 in regulating ORN axon targeting to the VA1d glomerulus (Figure 7B). *Pvf3* is expressed at a relatively high level in VA1d-PNs (Figures 7G and S8C). *Pvf3* knockdown in VA1d-PNs (and DC3/DA1-PNs) caused VA1d-ORNs to mistarget to the neighboring VA1v glomerulus (Figures 7C, 7D, and 7F). Indicating that Pvf3 is secreted by PNs to regulate ORN axon targeting, pan-ORN *Pvf3* RNAi did not cause significant targeting defects (Figure S9A). Intriguingly, overexpression of *Pvf3* in VA1d-PNs (and DC3/DA1-PNs) also caused VA1d-ORN axons to mistarget to the VA1v glomerulus (Figures 7E and 7F). Among VA1d’s neighbors, VA1v-PNs exhibited the most similar *Pvf3* expression to VA1d-PNs, albeit at a slightly lower level (Figure 7G). One hypothesis that could account for these observations is that Pvr directs ORN axons to glomeruli that express Pvf3 at a level most comparable with their original targets.

If Pvr mediates ORN responsiveness to Pvf3, then altering receptor levels should affect axon targeting. Indeed, VA1d-ORN-specific knockdown of *Pvr* caused increased targeting to the DA1 and VA1v glomeruli (Figures 7H, 7I, 7L, and 7M). Both glomeruli express lower levels of *Pvf3* than VA1d (Figure 7G), implying that when receptor levels are reduced, ORN axons target glomeruli with lower ligand expression. We therefore hypothesized that *Pvr* overexpression would enable VA1d-ORNs to innervate glomeruli in which ligand levels are higher than their original target. Thus, we generated a *Pvr* overexpression transgene (*UAS-Pvr*^*WT*^). However, elevated *Pvr* levels in VA1d-ORNs did not significantly alter axon target selection (Figures 7J and 7L–7P).

We hypothesized that ORNs may actively downregulate excess Pvr via endocytosis to maintain receptor levels needed to match the target-derived Pvf3. To test this, we identified two putative endocytic motifs flanking Pvr’s tyrosine kinase domain (Figure 7A) and generated a mutant transgene with these residues mutated to alanines (*UAS-Pvr*^*endo.mut*.^). When expressed in ORNs, both Pvr^WT^ and Pvr^endo.mut.^ trafficked to axon terminals (Figures 7Q and 7S). Consistent with the endocytome data in which Pvr is similarly distributed between the surface and endosomes (Figure 6D), Pvr^WT^ was present in both compartments (Figures 7Q and 7R). By contrast, Pvr^endo.mut.^ appeared mostly at the cell surface and did not strongly colocalize with endosomes (Figures 7S and 7T), a distribution also observed in cultured cells (Figure S10).

Interestingly, overexpression of endocytosis-deficient *Pvr* caused VA1d-ORN axons to mistarget to DC3 and VA5 glomeruli (Figures 7K and 7N–7P). Consistent with the matching-level hypothesis, both DC3 and VA5 glomeruli express *Pvf3* at higher levels than VA1d (Figure 7G). Finally, we probed the broader requirement for this signaling mechanism in DM6-ORNs and could recapitulate all *Pvr* loss- and gain-of-function phenotypes in these neurons (Figures S9B–S9G). Together, these data support the idea that clathrin-mediated endocytosis finely tunes axonal Pvr surface presentation to match Pvf3 levels in the target (Figure 7U). Altogether, these data reveal how developing neurons use endocytosis to actively modulate CSP localization and control axon target selection.

## DISCUSSION

### Endocytome profiling unlocks new dimensions of proteome remodeling in intact tissues

Proximity labeling-based proteomics has been used to define (1) how the surface proteome differs between distinct neural cell types,^62^ (2) the neuron-astrocyte proteomic interface,^63^ and (3) steady-state proteome changes over developmental time.^6,7^ Endocytome profiling achieves these goals and offers three key innovations. First, it transforms static snapshots of the surface proteome into a dynamic view of its remodeling at any given time *in situ*. Notably, our proteomes are derived from axons from a specific cell type, constituting less than 5% of the total axons in the fly brain—a major advance as such remodeling has, until now, been studied primarily in culture systems.^8–10^ Second, beyond capturing CSP dynamics, this approach also reveals transcellular communication by detecting proteins internalized from neighboring cells. Finally, it enables high-quality profiling of the endolysosomal network from sparse populations, overcoming potential limitations of other methods, which have thus far been restricted to bulk neural populations.^41,64,65^ This paves the way for uncovering how membrane trafficking varies across cell types or states and demonstrates that proximity labeling can be used for organelle-specific proteomics with cell-type resolution.

As was the case with cell-surface proteomics approaches in mammals,^7,62^ which we first established in *Drosophila*,^6^ we expect that endocytome profiling will be readily adapted for use in other model systems. The broad application of this tool is poised to reveal how the surface proteome is reconfigured to support activity-dependent morphological plasticity, to identify proteins transcytosed across the blood-brain barrier, or to uncover trafficking defects in disease models with endolysosomal dysfunction. This positions endocytome profiling as a powerful approach for dissecting cell-surface remodeling and membrane trafficking across diverse biological and pathological contexts.

### The endocytome reveals CSP regulation underlying neuronal connectivity

Endocytome profiling provided a systems-level view of how endocytosis actively sculpts the neuronal surface proteome to regulate circuit connectivity. Although this has been appreciated at the single-CSP level,^14–16,66^ our mechanistic studies elucidated that even within a single ORN type (DM6-ORNs) and time point, endocytosis regulates multiple CSPs to coordinate the developmental events necessary to build and maintain neural circuits.

This work revealed that developing axons may repurpose cell-cell junction proteins as part of a conserved developmental mechanism. Accordingly, a recent study found that transcripts encoding junctional proteins are enriched in central mammalian neurons during neurite outgrowth,^4^ and a *C. elegans* tight junction CSP regulates dendrite growth.^67^ In line with this, many endosome-enriched junctional proteins either had direct mammalian orthologs or were part of conserved families present across phyla. Our loss-of-function screen demonstrated that this class of CSPs broadly regulates axon development. Altogether, these findings indicate that junctional proteins may exhibit conserved neuronal functions and imply that their roles in circuit development are actively shaped by endocytic regulation.

As an example of such regulation, our analysis of crb both uncovered a new neuronal function and showed how endocytosis sculpts the surface proteome to meet stage-specific developmental needs. Specifically, crb promotes axon pruning during development but must be downregulated via endocytosis to prevent over-pruning of the mature terminal (Figure 5M). Sustained surface localization of crb destabilizes ORN axons, which contrasts its role in photoreceptors, in which *crb* loss leads to degeneration^51^ and *CRB1* and *CRB2* mutations are linked to retinal dystrophies in humans.^52^ Thus, while crb is essential for photoreceptor integrity, it must be tightly regulated in ORNs to preserve axon stability. Given its conserved retinal functions and the expression of *CRB1*/*CRB2* in the developing mammalian brain,^53,54^ it is possible that these orthologs also mediate neurite pruning in mammals.

A strength of cell-type-specific profiling is the ability to interrogate cell-cell interactions within intact tissues. Our analysis demonstrated that endocytome data can be integrated with complementary transcriptome profiling approaches to reveal transcellular signaling. In so doing, we discovered that an endosome-enriched growth factor, Pvf3, is secreted by postsynaptic partner PNs to regulate ORN axon target selection via its receptor, Pvr. Though *Pvf3* is expressed in most PN types, its differential expression is required for ORN axon targeting, raising the question of how a broadly expressed ligand can mediate ORN-type-specific outcomes. PDGF, Pvf3’s vertebrate ortholog, triggers a concentration-dependent switch between migration and proliferation by activating distinct signaling pathways downstream of the PDGF receptor.^68^ ORN axons transiently explore neighboring glomeruli before stabilizing in their target.^22,69^ Thus, varying postsynaptic Pvf3 levels could trigger different signaling responses in distinct ORN types, promoting retraction from incorrect glomeruli and stabilization in the correct target. Future work addressing the signaling outcomes downstream of Pvf3-Pvr activation will be important for fully understanding the role of these proteins in circuit assembly.

In summary, endocytome profiling provides a versatile framework for mapping surface proteome dynamics across cell types, developmental stages, and species. Our findings demonstrate how profiling CSP distribution in the native tissue environment reveals key principles of neuronal development and cellular organization. Broad application of this approach should not only expand our understanding of how the plasma membrane protein landscape is remodeled in health and disease but also uncover generalizable mechanisms of cellular plasticity.

### Limitations of the study

The limitations of HRP-based proximity labeling have been described elsewhere^70^; here we highlight considerations specific to our approach. First, whereas HRP^endo^ has high specificity for the endolysosomal compartment, it is not restricted to a distinct subset of vesicles, such as recycling endosomes or lysosomes. Thus, follow-up studies are needed to determine the trafficking fate(s) of internalized cargos. Second, HRP^endo^ relies on clathrin-dependent endocytosis, and while cargos internalized by alternative mechanisms could intersect with HRP^endo^ at sorting endosomes, this strategy could be less effective in cell types or contexts that primarily employ clathrin-independent uptake. Finally, a minimum number of cells per brain is likely required to express HRP^endo^ to generate a robust signal for mass spectrometry. Consequently, our proteomes are from all ORNs rather than distinct subtypes, which may obscure low-abundance or subtype-specific cargos. It is possible that utilizing labeling substrates not naturally found in samples, such as alkyne-phenol^71^ (in lieu of biotin-phenol), could improve the signal-to-noise ratio and enable profiling of lower-abundance cell types. Nevertheless, we anticipate that the increasing sensitivity of mass spectrometry will enable subtype-specific profiling in the future.

## RESOURCE AVAILABILITY

### Lead contact

Requests for further information, resources, and reagents generated by this study should be directed to the lead contact, Liqun Luo (lluo@stanford.edu).

### Materials availability

- *Drosophila* strains generated in this study will be deposited into the Bloomington Drosophila Stock Center.
- New DNA constructs will be deposited to Addgene.
- All other unique reagents generated in this study are available from the lead contact.

### Data and code availability

- The original mass spectra and the protein sequence databases used for searches have been deposited in the public proteomics repository MassIVE (http://massive.ucsd.edu) and are accessible at ftp://MSV000097831@massive-ftp.ucsd.edu. Processed proteomic data are provided in Table S2.
- This paper does not report original code.
- Any additional information required to reanalyze the data reported in this paper is available from the lead contact upon request.

## STAR★METHODS

### EXPERIMENTAL MODEL AND STUDY PARTICIPANT DETAILS

#### *Drosophila* stocks and genotypes

Flies were maintained on standard cornmeal media with a 12 h light-dark cycle at 25°C, except for RNAi/overexpression crosses which were raised at 29°C. Complete genotypes for flies used in each experiment are described in Table S1.

We note that *Peb-GAL4*, the driver used to express UAS-HRP transgenes, is present in neurons in the visual and auditory systems as well as ~20 other neurons in the brain. Our dissections removed the visual system but not the other neurons. However, they comprise less than 10% of the axons profiled and thus represent a minor fraction of our proteomes.

### METHOD DETAILS

#### Generation of UAS constructs and transgenic flies

*UAS-HA-HRP*^*surf*^ and *UAS-HA-HRP*^*endo*^ flies were generated from a gBlock containing a wingless signal peptide upstream of an epitope tag (HA) followed by HRP fused to the N terminal regions of human CD2. The gBlock was cloned into a 5x *pUASt-attB* vector. The cytoplasmic tail of CD2 was mutated via site-directed mutagenesis (New England Biolabs) to include a dileucine endocytic motif or a motif consisting of all alanines. Aside from the addition of the HA tag and dileucine motif (or alanines), these transgenes are otherwise identical to the one used in Li et al.^6^ The constructs were validated by full-length plasmid sequencing and injected into embryos bearing the *attP24* landing site. G0 flies were crossed to a white– balancer, and all white+ progeny were individually balanced.

*UAS-Pvf3-myc, UAS-Pvr*^*WT*^*-V5, UAS-Pvr*^*endo.mut*.^*-V5, UAS-2xFYVE-mCherry* flies were synthesized and cloned by Twist Biosciences into a 10x *pUASt-attB* vector. cDNA sequences for the isoforms of Pvf3 (isoform D) and Pvr (isoform J) identified in our proteomics were used to generate each rescue transgene. For the *UAS-Pvr*^*endo.mut*.^*-V5* transgene, all residues in the endocytic motifs were mutated to alanines with the exception of tyrosine (Y) residue in the second motif which was left intact to minimally disrupt signaling. Epitope tags are C-terminal just prior to the stop codon. 2xFYVE-mCherry was fly codon optimized. The constructs were all validated full-length plasmid sequencing and injected into embryos bearing an attP40 (*UAS-2xFYVE-mCherry*,), attP2 (*UAS-Pvf3-myc*), or attP86Fb (*UAS-Pvr* constructs, *UAS-2xFYVE-mCherry*). G0 flies were crossed to a white– balancer and all white+ progeny were individually balanced.

All flies were injected in-house using standard microinjection methods, except *UAS-Pvr*^*WT*^*-V5* which was made through Best Gene Inc.

#### HRP-mediated proximity biotinylation of proteins in ORN endosomes and surface

Proximity labeling was performed using previously published methods^6^ with one key difference in the usage of the membrane-permeable biotin-phenol instead of membrane-impermeable BXXP as a substrate which enables the labeling of proteins in the intracellular compartments in addition to just extracellular or surface proteins. Briefly, 30–36 h APF (after puparium formation) brains containing ORN undergoing axon target selection were dissected (optic lobes were removed) in ice cold Schneider’s media (ThermoFisher) and transferred into a 1.5 mL protein low-binding tube (Eppendorf) containing additional media on ice. Brains were washed with Schneider’s media to remove debris and incubated in 500 μM biotin-phenol (BP; APExBio) in Schneider’s media while rotating at 4°C for 1 h. Brains were subsequently labeled with 1 mM (0.003%) H_2_O_2_ (ThermoFisher) for 10 min while rotating and immediately quenched by five thorough washes using the quenching buffer (10 mM sodium ascorbate, 5 mM Trolox, and 10 mM sodium azide in phosphate buffered saline [PBS]). Following these washes, the quenching solution was removed, and brains were either fixed for immunostaining (see below for details) or were snap frozen and stored at −80°C for downstream processing for proteomic analysis.

#### Enrichment of biotinylated proteins

Brains were processed in the original collection tube to avoid loss during transferring using previously published methods.^6,69,89^ Briefly, 40 μL of high-SDS RIPA buffer (50 mM Tris-HCl [pH 8.0], 150 mM NaCl, 1% sodium dodecyl sulfate [SDS], 0.5% sodium deoxycholate, 1% Triton X-100, 1x protease inhibitor cocktail [Sigma-Aldrich], and 1 mM phenylmethylsulfonyl fluoride [PMSF; Sigma-Aldrich]) was added to each tube and frozen brains were homogenized on ice. Next, samples of the same experimental group were spun down and merged, rinsed with additional 100 μL of high-SDS RIPA, vortexed, and sonicated briefly. Lysates were diluted with 1.2 mL of SDS-free RIPA buffer (50 mM Tris-HCl [pH 8.0], 150 mM NaCl, 0.5% sodium deoxycholate, 1% Triton X-100, 1x protease inhibitor cocktail, and 1 mM PMSF) and rotated for 1 h at 4°C. Lysates were then diluted with 200 μL of normal RIPA buffer (50 mM Tris-HCl [pH 8.0], 150 mM NaCl, 0.2% SDS, 0.5% sodium deoxycholate, 1% Triton X-100, 1x protease inhibitor cocktail, and 1 mM PMSF), transferred to a 3.5 mL ultracentrifuge tube (Beckmann Coulter), and centrifuged at 100,000 × g for 30 min at 4°C. 1.5 mL of supernatant was collected for each sample and added to 210 μL of pre-washed streptavidin magnetic beads (Pierce) and incubated while rotating at 4°C overnight. The next day, beads were washed twice with 1 mL RIPA buffer, once with 1 mL of KCl, then with 1mL 0.1 M Na_2_CO_3_, followed by 1 mL 2 M urea (in 10 mM Tris-HCl [pH 8.0]), and finally twice with 1 mL RIPA buffer.

Finally, beads were washed twice in 1 mL NaCl (75 mM NaCl in 50 mM Tris HCl [pH 8]) buffer and resuspended in 400 μL of NaCl buffer. 10% of the bead suspension was removed for western blot analysis, and the rest were snap frozen prior to on-bead digestion.

#### Western blotting of biotinylated proteins

Biotinylated proteins were eluted from streptavidin beads via the addition of 20 μL of elution buffer (2X Laemmli sample buffer [BioRad], 20 mM dithiothreitol [Sigma-Aldrich], and 2mM biotin [Sigma-Aldrich]) followed by a 10 min incubation at 95°C. Proteins were loaded on to 4%–12% Bis-Tris PAGE gels (ThermoFisher) and transferred to PVDF membranes (ThermoFisher). After blocking with Intercept (TBS) blocking buffer (LI-COR) for 1 h, membranes were incubated with Streptavidin-800 (LI-COR) and visualized using the ChemiDoc imaging system (BioRad).

#### On-bead trypsin digestion of biotinylated proteins

Peptides bound to streptavidin magnetic beads were washed four times with 200 μL of 50 mM Tris-HCl (pH = 7.5) buffer. After removing the final wash, the beads were incubated twice at room temperature (RT) in 80 μL of the digestion buffer – 2 M Urea, 50 mM Tris-HCl, 1 mM DTT, and 0.4 μg trypsin – while shaking at 1000 rpm. The first incubation lasted 1 h, followed by the second incubation of 30 min. After each incubation, the supernatant was collected and transferred into a separate tube. The beads were then washed twice with 60 μL of 2 M Urea/ 50 mM Tris-HCl buffer. The resulting washes were combined with the digestion supernatant. The pooled eluate of each sample was then spun down at 5000 × g for 30 s to collect the supernatant. The samples were subsequently reduced with 4 mM DTT for 30 min at RT with shaking at 1000 rpm, followed by alkylation with 10 mM Iodoacetamide for 45 min in the dark at RT while shaking at 1000 rpm. Overnight digestion of the samples was performed by adding 0.5 μg of trypsin to each sample. The following morning, the samples were acidified with neat formic acid (FA) to the final concentration of 1% FA (pH<3).

Digested peptide samples were desalted using in-house packed C18 (3M) StageTips. C18 StageTips were conditioned sequentially with 100 μL of 100% methanol (MeOH), 100 μL of 50% (vol/vol) acetonitrile (MeCN) with 0.1% (vol/vol) FA, and two washes of 100 μL of 0.1% (vol/vol) FA. Acidified peptides were loaded onto the C18 StageTips and washed twice with 100 μL of 0.1% FA. The peptides were then eluted from the C18 resin using 50 μL of 50% MeCN/ 0.1% FA. The desalted peptide samples were snap-frozen and vacuum-centrifuged until completely dry.

#### TMT labeling and stagetip peptide fractionation

Desalted peptides were labeled with TMT16 reagents (Thermo Fisher Scientific). For this experiment, relevant TMT channels are 131N-ORN_NC 1, 132C-ORN_NC 2, 128C-ORN_HRP^endo^ 1, 133N-ORN_ HRP^endo^ 2, 133C-ORN_ HRP^endo^ 3, 129C-ORN_ HRP^surf^ 1, 130N-ORN_ HRP^surf^ 2, 127C-ORN_ HRP^surf^ 3. Each peptide sample was resuspended in 80 μL of 50 mM HEPES and labeled with 20 μL of the 25 μg/μL TMT reagents in MeCN. The samples were then incubated at RT for 1 h while shaking at 1000 rpm. To quench the TMT-labeling reaction, 4 μL of 5% hydroxylamine was added to each sample, followed by a 15-min incubation at RT with shaking. TMT-labeled samples were combined and vacuum-centrifuged to dry. The samples were then reconstituted in 200 μL of 0.1% FA and desalted on a C18 StageTip using the previously described protocol. The desalted TMT-labeled combined sample was then dried to completion.

The combined TMT-labeled peptide sample was fractionated by basic reverse phase (bRP) fractionation using an in-house packed SDB-RPS (3M) StageTip. A StageTip containing three plugs of SDB material was prepared and conditioned with 100 μL of 100% MeOH, 100 μL of 50% MeCN/0.1% FA, and 2x with 100 μL of 0.1% FA. The sample was resuspended in 200 μL 0.1% FA (pH < 3) and loaded onto the conditioned StageTip and eluted in a series of buffers with increasing MeCN concentrations. Eight fractions were collected in 20 mM ammonium formate (5%, 7.5%, 10%, 12.5%, 15%, 20%, 25%, and 45% MeCN), dried to completion and analyzed by LC-MS/MS.

#### Liquid chromatography and mass spectrometry

All peptide samples were separated and analyzed on an online liquid chromatography tandem mass spectrometry (LC-MS/MS) system, consisting of a Vanquish Neo UPHLC (Thermo Fisher Scientific) coupled to an Orbitrap Exploris 480 (Thermo Fisher Scientific). All peptide fractions were reconstituted in 9 μL of 3% MeCN/ 0.1% FA. 4 μL of each fraction was injected onto a microcapillary column (Picofrit with 10 μm tip opening/ 75 μm diameter, New Objective, PF360-75-10-N-5), packed in-house with 30 cm of C18 silica material (1.5 μm ReproSil-Pur C18-AQ medium, Dr. Maisch GmbH, r119.aq) and heated to 50°C using column heater sleeves (PhoenixST). Peptides were eluted into the Orbitrap Exploris 480 at a flow rate of 200 nL/min. The bRP fractions were run on a 154 min-method. Solvent A comprised 3% acetonitrile/ 0.1% FA. Solvent B comprised 90% acetonitrile/ 0.1% FA. The LC-MS/MS method used the following gradient profile: (min: %B) 0:2; 1:6; 122:35; 130:60; 133:90; 143:90; 144:50; 154:50 (the last two steps at 500 nl/min flow rate).

Mass spectrometry was conducted using a data-dependent acquisition mode, MS1 spectra were measured with a resolution of 60,000, a normalized AGC target of 100%, and a mass range from 350 to 1800 m/z. MS2 spectra were acquired for the top 20 most abundant ions per cycle at a resolution of 45,000, an AGC target of 50%, an isolation window of 0.7 m/z and a normalized collision energy of 32. The dynamic exclusion time was set to 20 s.

#### Mass spectrometry data processing

Mass spectrometry data was processed using Spectrum Mill (proteomics.broadinstitute.org). Spectra within a precursor mass range of 600–6000 Da with a minimum MS1 signal-to-noise ratio of 25 were retained. Additionally, MS1 spectra within a retention time range of ± 45 s, or within a precursor m/z tolerance of ± 1.4 m/z were merged. MS/MS searching was performed against a human Uniprot database. For searching, fixed modifications were TMT16-Full-Lys modification and carbamidomethylation on cysteine. Variable modifications included acetylation of the protein N-terminus, oxidation of methionine and cyclization to pyroglutamic acid. Digestion parameters were set to “trypsin allow P” with an allowance of 4 missed cleavages. The matching tolerances were set with a minimum matched peak intensity of 30%, precursor and product mass tolerance of ± 20 ppm.

#### Quantitative comparison of endosome and surface proteomes

Peptide spectrum matches (PSMs) were validated with a maximum false discovery rate (FDR) threshold of 1.2% for precursor charges ranging from +2 to +6. A target protein score of 9 was applied during protein polishing auto-validation to further filter PSMs. TMT16 reporter ion intensities were corrected for isotopic impurities using the afRICA correction method in the Spectrum Mill protein/ peptide summary module, which utilizes determinant calculations according to Cramer’s Rule. Protein quantification and statistical analysis were performed using the Proteomics Toolset for Integrative Data Analysis (Protigy, v1.0.7, Broad Institute, https://github.com/broadinstitute/protigy). Differential protein expression was evaluated using moderated *t* tests, with *p* values calculated to assess significance. Note: TMT-based proteomics can have ratio compression and result in an underestimation of true abundance differences between samples.

#### Proteomic data cutoff analysis

We used a ratiometric strategy^30^ to remove contaminants. Briefly, all detected proteins were annotated as either true-positives (TPs; proteins with a signal peptide and/or a transmembrane domain) or false-positives (FPs; those without either domain) according to the UniProt database. For each experimental group, we calculated the TMT ratios of proteins within this group compared to an averaged negative control value and sorted proteins in descending order. For each TMT ratio, a true-positive rate (TPR) and false positive rate (FPR) were calculated by adding the number of TPs or FPS with a higher ranking and dividing them by the total number of TPs and FPs, respectively. The TPRs and FPRs were used to generate the ROC curve. Cutoffs for experimental groups were determined by finding the TMT ratio where [TPR – FPR] is maximized. Proteins with TMT ratios higher than the cutoff in each experimental group were retained. Finally, proteins present in all three experimental groups were retained for downstream analysis.

#### Immunostaining

Fly brains were dissected according to a previously published protocol.^90^ In brief, brains were dissected in PBS, transferred to a tube containing 4% paraformaldehyde in PBST (0.3% Triton X-100), and fixed for 20 min while nutating at RT. Following fixation, brains were washed 3 times for 20 min in PBST and blocked for at least 30 min in PBST + 5% normal donkey serum. The following antibodies were used: rat anti-Ncad (Developmental Studies Hybridoma Bank; 1:40), chicken anti-GFP (Aves Labs; 1:1000), rabbit anti-HA (Cell Signaling Technologies; 1:200), rat anti-HA (Sigma Aldrich; 1:200), mouse anti-V5 (ThermoFisher Scientific; 1:100), guinea pig anti-Mcr^72^; (1:500), rabbit anti-dsRed (Takara Bio; 1:1000), rabbit anti-Lamp1 (Abcam; 1:100), rabbit anti-Rab5 (Abcam; 1:100), mouse anti-Rab7 (Developmental Studies Hybridoma Bank; 1:10), goat anti-HRP-594 (Jackson ImmunoResearch; 1:300), mouse anti-mCherry (ThermoFisher Scientific; 1:1000), mouse anti-Rab11^91^ (BD Biosciences; 1:100), rabbit anti-Rab5^92^ (Abcam; 1:100), and rat anti-V5 (Abcam; 1:500) and incubated with brains in block buffer overnight at 4°C while nutating. Brains were subsequently washed three times for 20 min in PBST and incubated in secondary antibodies (Alexa Fluor 488; Alexa Fluor 564; Alexa Fluor 647; 1:200) overnight at 4°C while nutating. Brains were again washed three times for 20 min in PBST, transferred to SlowFade antifade reagent (ThermoFisher) and stored at 4°C prior to mounting. Neutravidin-647 (ThermoFisher) was used to visualize biotinylated proteins.

#### GFP ELISA

ELISAs for GFP in flies pan-neuronally expressing UAS-crb transgenes were performed on one L3 central nervous system according to the manufacturers protocol (Abcam ab171581) with one key difference: whole brain lysates were prepared in PBS + 0.1% Tween according to Jay et al.^93^

#### Image acquisition and processing

Images were obtained on a Zeiss LSM900 laser-scanning confocal microscope (Carl Zeiss) using either a 40x oil immersion objective (axon targeting experiments) or a 63x oil immersion objective (Airyscan experiments). 16-bit z-stacks for axon targeting experiments were acquired at 1 μm intervals at a resolution of 1024 × 1024. Airyscan images were taken at software optimized resolution and intervals. Brightness and contrast adjustments as well as image cropping was done using Photoshop or Illustrator (Adobe).

#### RNAi-based genetic screen

The AM29 screening line was generated by converting *AM29-GAL4* to *AM29-QF2* via the homology assisted CRISPR knock-in (HACK) method.^94^ *AM29-QF2* was recombined with *QUAS-mtdTomato* on the second chromosome and put with *Peb-GAL4, UAS-dcr2* on the X chromosome. Virgin females from the AM29 screening line were crossed to *UAS-RNAi* males and the progeny were kept at 25°C for 2–5 days following egg laying and then transferred to 29°C to enhance transgene expression. Brains were dissected, processed, and imaged as described above. *Peb-GAL4* is expressed in some peripheral tissues and expression of some RNAi lines using this driver caused adult lethality. To circumvent this, we dissected late-stage pupae of these genotypes and evaluated their axon targeting.

For this analysis, we identified glomeruli using NCad labeling (based on the stereotypy of their size, shape, and positions) and categorized the extent of innervation into each glomerulus (not innervated; weakly innervated; strongly innervated). Axon targeting analysis was performed blinded to genotype when possible. Data was analyzed using Prism10 (GraphPad). For each glomerulus, we calculated the frequency of each type of innervation and plotted the results as stacked bar charts. Fisher’s exact test was performed on innervation frequencies in each glomerulus to determine statistical significance compared to controls. *p* values were adjusted using the Bonferroni correction.

#### *shibire*^*ts*^ temperature shift paradigm

*AM29-GAL4* virgins were crossed to males bearing *UAS-shi*^*ts*^ and allowed to reproduce at room temperature for 2–5 days. Since rearing flies at temperatures different from 25°C (standard rearing temperature) affects developmental progression, we interpolated the developmental timepoint of flies at each temperature (18°C or 30°C) using published developmental progression data.^95^ Progeny were moved to 18°C (restrictive temperature) until pupariation. Once pupae formed, 0–6 h pupae were collected and allowed to develop until they were ~24 h APF. At this time, flies were moved to a 30°C incubator for 24 h. Pupae were then returned to 18°C and allowed to develop into adults when their brains were dissected, processed, and analyzed for mistargeting as described above. Analysis was performed blinded to experimental manipulation. Control flies were the same genotype as experimental ones but reared exclusively at 18 °C.

#### MARCM-based mosaic analysis

*hs-FLP* based MARCM analyses were performed by heat shocking 0–24h APF pupae for 1 h at 37°C. Each fly contained *AM29-GAL4, UAS-mCD8-GFP, UAS-mtdTomato, tubP-Gal80*, the desired *FRT* site, a mutant allele distal to the *FRT* site (or no allele for control), and in rescue experiments a *UAS-crb* rescue transgene (see Table S1 for complete genotypes). *eyFLP* based MARCM analyses were not heat shocked but contained the same genomic components as *hs-FLP* MARCM flies. Brains were dissected, processed, and mounted as described above. MARCM phenotypes were analyzed blinded to genotype and a Fisher’s exact test was performed on MARCM phenotypes. *p* values were adjusted using the Bonferroni correction.

#### Transfection and immunostaining of *Drosophila* S2 cells

S2 cells were co-transfected with *Actin-GAL4* and either *pUASt-attB-Pvr*^*WT*^*-V5, pUASt-attB-Pvr*^*endo.mut*.^*-V5, pUASt-attB-HA-HRP*^*surf*^*, or pUASt-attB-HA-HRP*^*endo*^ using Effectine (Qiagen). After 48 h, transfected cells were washed in 1X PBS, fixed in 4% PFA in PBS for 15 min, permeabilized in PBST, blocked in 5% normal donkey serum in PBST, and stained with: rabbit anti-HA (C29F4; Cell Signaling Technology) or rat anti-HA (3F10; Sigma Aldrich), mouse anti-Rab7 (Developmental Studies Hybridoma Bank), rabbit anti-Rab5 (Abcam); or mouse anti-V5 (ThermoFisher) or rabbit anti-V5 (ThermoFisher) at room temperature for 2 h, followed by three 15 min washes in PBST, and incubated in secondary antibody for 2 h at room temperature.

For antibody internalization assays, cells were transfected as previously described. After 48 h, transfected cells were washed in 1X PBS and anti-HRP-594 was added to the media at 1:100. Cells were incubated at room temperature in media for 30, 60, or 120 min, washed in PBS, fixed in 4% PFA in PBST, washed again three times in PBST for 15 min each, and imaged. The number of internalized HRP-594 puncta were quantified and normalized to the area of each cell.

#### Gene Ontology and STRING network analysis

Gene Ontology (GO) analysis was performed using Flymine. We minimized GO redundancy using REVIGO.^88^ The STRING database was used to determine protein-protein interactions in Figure 4A, and nodes were connected based on experimental data. GO terms in chord and STRING plots were determined using Flymine. Specifically, we selected each term based on 1) breadth (we displayed broader terms); 2) experimental validation; and 3) neurodevelopmental relevance (we prioritized terms in this category). We displayed GO categories on these plots that had two or more proteins within them.

To generate the diagram in Figure 2E we compiled a list of proteins that have been shown to function in the endolysosomal system in mammalian cells^8,96^ as well as in *Drosophila*. Since there has not been comprehensive profiling of the fly endolysosomal pathway we used Flybase (www.flybase.org) cellular component search tool to identify such proteins. For the mammalian proteins, we utilized the Flybase homologs search tool to identify the *Drosophila* homologs of mammalian proteins.

### QUANTIFICATION AND STATISTICAL ANALYSIS

The statistical tests and numbers of independent replicates per experiment are indicated in the figures or figure legends.

#### Single-cell RNA-seq analysis

scRNA-seq data is from McLaughlin et al.^3^ (ORNs) and Xie et al.^5^ (PNs). For analysis in Figure 6, we averaged expression from the two developmental time points (24 h APF and 48 h APF) bracketing our profiling (36 h APF) and from all ORN or PN subtypes, including clusters that have not yet been mapped to a specific ORN or PN subtype. A transcript was considered enriched if it was expressed in > 30% of cells at log_2_(CPM+1) ≥ 4. We note that a few transcripts that are only expressed in a few cell types or are very lowly expressed (Figure S6) may be false negatives in this analysis.

Analysis of human neuronal expression of orthologs for the top 200 TM proteins labeled by endosome HRP in Table S3 is based on data in Hodge et al.^97^

#### Ligand-receptor pair and human ortholog identification

Potential ligand-receptor pairs were identified using interaction information on Flybase (www.flybase.org) and through literature searches. Human orthologs were identified using the Flybase Homolog Search function and only orthologs with a score > 3 were displayed. For simplicity, in most cases, we only displayed the ortholog(s) with the ‘Best Score’.

#### Statistical analysis

Statistical tests for mutant phenotypic quantification are as follows. For categorical data we used a Fisher’s exact test followed by a Bonferroni correction to determine statistical significance. To determine statistical significance between continuous data sets, we used Mann-Whitney test for comparisons between two groups or a Kruskal-Wallis test (followed by Dunn’s multiple comparisons test) for comparisons between multiple groups. Error bars in all plots represent standard error of the mean (SEM).

#### Quantification of *Pvf3* expression in the antennal lobe

*Pvf3-GAL4* was crossed to *GH146-FLP; UAS>stop>mCD8-GFP* flies. Flies were reared at 25°C and 42–48 h APF pupae were dissected, labeled, and imaged according to the descriptions above. Glomeruli were identified based on stereotyped location and shape and fluorescence intensity in the brightest section of each glomerulus was measured. Background values were subtracted from intensity measurements and fluorescence intensity in each glomerulus was normalized to the average intensity in each antennal lobe. Fluorescence intensity of each glomerulus across all brains measured was averaged, and a heatmap was applied to each value. 3D volume renderings were created in Imaris10 (Oxford Instruments) and the heatmap values were transferred onto each glomerulus.

## Supplementary Material

MMC3

MMC2

MMC1

Supplemental information can be found online at https://doi.org/10.1016/j.neuron.2026.01.027.

## Figures and Tables

**Table:** KEY RESOURCES TABLE

REAGENT or RESOURCE	SOURCE	IDENTIFIER
Antibodies		
Rat anti-Ncad	Developmental Studies Hybridoma Bank	Cat# DN-Ex #8; RRID: AB_528121
Chicken anti-GFP	Aves labs	Cat# GFP-1020; RRID: AB_10000240
Rabbit anti-HA	Cell Signaling Technologies	Cat# 3724; RRID: AB_1549585
Rat anti-HA	Sigma Aldrich	RRID: AB_10094468
Mouse anti-V5	Thermofisher Scientific	Cat# R960-25; RRID: AB_2556564
Guinea pig anti-Mcr	Hall et al.^72^	N/A
Rabbit anti-dsRed	Takara Bio	Cat# 632496; RRID: AB_10013483
Rabbit anti-Lamp1	Abcam	RRID:AB_775973
Rabbit anti-Rab5	Abcam	RRID:AB_882240
Mouse anti-Rab7	Developmental Studies Hybridoma Bank	RRID:AB_2722471
Gpat anti-HRP-594	Jackson ImmunoResearch	RRID:AB_2338966
Mouse anti-mCherry	Thermofisher Scientific	RRID: AB_2536611
Rat anti-V5	Abcam	Cat# ab206571
Mouse anti-Rab11	BD Biosciences	AB_397984
Mouse anti-Fas3	Developmental Studies Hybridoma Bank	Cat# 7G10; RRID:AB_528238
Mouse anti-Nrg	Developmental Studies Hybridoma Bank	Cat# BP 104; RRID:AB_528402
Deposited data		
Mass spectrometry proteomics data	This paper	N/A
Experimental models: Organisms/strains		
D. melanogaster: tubP-GAL80	Lee and Luo^56^	RRID: BDSC_9917
D. melanogaster: UAS-mCD8-GFP	Lee and Luo^56^	RRID: DGRC_108068
D. melanogaster: hsFlp	Golic and Lindquist^73^	N/A
D. melanogaster: FRT82B	Xu and Rubin^74^	N/A
D. melanogaster: eyFLP	Newsome et al.^75^	N/A
D. melanogaster: crb11A22	Tepass and Knust^57^	RRID:BDSC_3448
D. melanogaster: UAS-crbWT-GFP	Simões et al.^58^	N/A
D. melanogaster: UAS-crbRR-GFP	Simões et al.^58^	N/A
D. melanogaster: TI{TI}crbGFP-C	Huang et al.^55^	N/A
D. melanogaster: AM29-GAL4	Endo et al.^76^	N/A
D. melanogaster: AM29-QF2	This paper	N/A
D. melanogaster: Peb-GAL4	Sweeney et al.^77^	N/A
D. melanogaster: UAS-Dcr-2	Dietzl et al.^78^	N/A
D. melanogaster: 20XUAS-IVS-shits1-p10	Pfeiffer et al.^79^	N/A
D. melanogaster: OK6-GAL4	Aberle et al.^80^	N/A
D. melanogaster: UAS-HRPendo	This paper	N/A
D. melanogaster: UAS-HRPsurf	This paper	N/A
D. melanogaster: UAS-Pvrwt-V5	This paper	N/A
D. melanogaster: UAS-Pvrendo.mut.-V5	This paper	N/A
D. melanogaster: UAS-Pvf3-myc	This paper	N/A
D. melanogaster: QUAS-mtdtomato-3xHA	Potter et al.^81^	RRID:BDSC: 30004
D. melanogaster: UAS-2xFYVE-mCherry	This paper	N/A
D. melanogaster: GH146-FLP	Hong et al.^82^	RRID:BDSC_81291
D. melanogaster: UAS-FRT-stop-FRT-mCD8-GFP	Potter et al.^81^	RRID:BDSC_30125
D. melanogaster: Mz19-GAL4	Ito et al.^83^	RRID:BDSC: 34497
D. melanogaster: Or88a-mtdTomato	N/A	N/A
D. melanogaster: Or47b-rCD2	Zhu and Luo^84^	RRID:BDSC: 9916
D. melanogaster: GMR78H05-p65AD	Dionne et al.^85^	RRID:BDSC: 601815
D. melanogaster: GMR31F09-GAL4DBD	Dionne et al.,^85^	RRID:BDSC: 68759
D. melanogaster: RNAi lines	Dietzl et al.^78^, Ni et al.^86^, and Perkins et al.^87^	Stock numbers are in Table S1
D. melanogaster: S2 cells	Thermofisher Scientific	Cat# R69007
Chemicals, peptides, and recombinant proteins		
Schneider’s Drosophila medium	Thermofisher Scientific	Cat# 21720001
Fetal Bovine Serum, heat inactivated	Thermofisher Scientific	Cat# A3840101
GFP ELISA Kit	Abcam	Cat# ab171581
Software and algorithms		
Zen Blue	Carl Zeiss	RRID: SCR_013672
Fiji	National Institutes of Health	RRID:SCR_002285
Illustrator	Adobe	RRID: SCR_010279
Python	N/A	RRID:SCR_008394
Spectrum Mill	Agilent	https://proteomics.broadinstitute.org
Flymine	N/A	RRID:SCR_002694
Revigo	Supek et al.^88^	RRID:SCR_005825
STRING	N/A	RRID:SCR_005223

## Highlights

- Endocytome profiling reveals rapid proteome remodeling by endocytosis in fly brains
- Cell-type-specific proteomics charts subcellular residence of over 1,000 proteins
- Axon refinement requires a precise endosome-to-surface ratio of a junctional protein
- Multi-omic integration uncovers transcellular signaling for neural circuit assembly

## References

[1] Kurmangaliyev YZ, Yoo J, Valdes-Aleman J, Sanfilippo P, and Zipursky SL (2020). Transcriptional Programs of Circuit Assembly in the Drosophila Visual System. Neuron 108, 1045–1057.e6. 10.1016/j.neuron.2020.10.006. 33125872

[2] Özel MN, Simon F, Jafari S, Holguera I, Chen Y-C, Benhra N, El-Danaf RN, Kapuralin K, Malin JA, Konstantinides N, (2021). Neuronal diversity and convergence in a visual system developmental atlas. Nature 589, 88–95. 10.1038/s41586-020-2879-3. 33149298 PMC7790857

[3] McLaughlin CN, Brbić M, Xie Q, Li T, Horns F, Kolluru SS, Kebschull JM, Vacek D, Xie A, Li J, (2021). Single-cell transcriptomes of developing and adult olfactory receptor neurons in Drosophila. eLife 10, e63856. 10.7554/elife.63856. 33555999 PMC7870146

[4] Gao Y, van Velthoven CTJ, Lee C, Thomas ED, Mathieu R, Ayala AP, Barta S, Bertagnolli D, Campos J, Cardenas T, (2025). Continuous cell-type diversification in mouse visual cortex development. Nature 647, 127–142. 10.1038/s41586-025-09644-1. 41193844 PMC12589121

[5] Xie Q, Brbic M, Horns F, Kolluru SS, Jones RC, Li J, Reddy AR, Xie A, Kohani S, Li Z, (2021). Temporal evolution of single-cell transcriptomes of Drosophila olfactory projection neurons. eLife 10, e63450. 10.7554/elife.63450. 33427646 PMC7870145

[6] Li J, Han S, Li H, Udeshi ND, Svinkina T, Mani DR, Xu C, Guajardo R, Xie Q, Li T, (2020). Cell-Surface Proteomic Profiling in the Fly Brain Uncovers Wiring Regulators. Cell 180, 373–386.e15. 10.1016/j.cell.2019.12.029. 31955847 PMC7072036

[7] Shuster SA, Li J, Chon U, Sinantha-Hu MC, Luginbuhl DJ, Udeshi ND, Carey DK, Takeo YH, Xie Q, Xu C, (2022). In situ cell-type-specific cell-surface proteomic profiling in mice. Neuron 110, 3882–3896.e9. 10.1016/j.neuron.2022.09.025. 36220098 PMC9742329

[8] Itzhak DN, Tyanova S, Cox J, and Borner GH (2016). Global, quantitative and dynamic mapping of protein subcellular localization. eLife 5, e16950. 10.7554/elife.16950. 27278775 PMC4959882

[9] Schessner JP, Albrecht V, Davies AK, Sinitcyn P, and Borner GHH (2023). Deep and fast label-free Dynamic Organellar Mapping. Nat. Commun 14, 5252. 10.1038/s41467-023-41000-7. 37644046 PMC10465578

[10] Hein MY, Peng D, Todorova V, McCarthy F, Kim K, Liu C, Savy L, Januel C, Baltazar-Nunez R, Sekhar M, (2025). Global organelle profiling reveals subcellular localization and remodeling at proteome scale. Cell 188, 1137–1155.e20. 10.1016/j.cell.2024.11.028. 39742809

[11] Gaidarov I, Santini F, Warren RA, and Keen JH (1999). Spatial control of coated-pit dynamics in living cells. Nat. Cell Biol 1, 1–7. 10.1038/8971. 10559856

[12] Villaseñ or R, Kalaidzidis Y, and Zerial M (2016). Signal processing by the endosomal system. Curr. Opin. Cell Biol 39, 53–60. 10.1016/j.ceb.2016.02.002. 26921695

[13] von Zastrow M, and Sorkin A (2021). Mechanisms for Regulating and Organizing Receptor Signaling by Endocytosis. Annu. Rev. Biochem 90, 709–737. 10.1146/annurev-biochem-081820-092427. 33606955 PMC8608402

[14] Scott-Solomon E, and Kuruvilla R (2018). Mechanisms of neurotrophin trafficking via Trk receptors. Mol. Cell. Neurosci 91, 25–33. 10.1016/j.mcn.2018.03.013. 29596897 PMC6128733

[15] Pasterkamp RJ, and Burk K (2021). Axon guidance receptors: Endocytosis, trafficking and downstream signaling from endosomes. Prog. Neurobiol 198, 101916. 10.1016/j.pneurobio.2020.101916. 32991957

[16] Sullivan KG, and Bashaw GJ (2023). Intracellular Trafficking Mechanisms that Regulate Repulsive Axon Guidance. Neuroscience 508, 123–136. 10.1016/j.neuroscience.2022.07.012. 35863679 PMC9839465

[17] Eichel K, Uenaka T, Belapurkar V, Lu R, Cheng S, Pak JS, Taylor CA, Südhof TC, Malenka R, Wernig M, (2022). Endocytosis in the axon initial segment maintains neuronal polarity. Nature 609, 128–135. 10.1038/s41586-022-05074-5. 35978188 PMC9433327

[18] Hinshaw JE, and Schmid SL (1995). Dynamin self-assembles into rings suggesting a mechanism for coated vesicle budding. Nature 374, 190–192. 10.1038/374190a0. 7877694

[19] Takei K, McPherson PS, Schmid SL, and De Camilli P (1995). Tubular membrane invaginations coated by dynamin rings are induced by GTP-γS in nerve terminals. Nature 374, 186–190. 10.1038/374186a0. 7877693

[20] Lyu C, Li Z, Xu C, Wong KKL, Luginbuhl DJ, McLaughlin CN, Xie Q, Li T, Li H, and Luo L (2025). Dimensionality reduction simplifies synaptic partner matching in an olfactory circuit. Science 388, 538–544. 10.1126/science.ads7633. 40310920 PMC12614222

[21] Koenig JH, Kosaka T, and Ikeda K (1989). The relationship between the number of synaptic vesicles and the amount of transmitter released. J. Neurosci 9, 1937–1942. 10.1523/jneurosci.09-06-01937.1989. 2566663 PMC6569730

[22] Li T, Fu T-M, Wong KKL, Li H, Xie Q, Luginbuhl DJ, Wagner MJ, Betzig E, and Luo L (2021). Cellular bases of olfactory circuit assembly revealed by systematic time-lapse imaging. Cell 184, 5107–5121.e14. 10.1016/j.cell.2021.08.030. 34551316 PMC8545656

[23] Aimino MA, DePew AT, Restrepo L, and Mosca TJ (2023). Synaptic Development in Diverse Olfactory Neuron Classes Uses Distinct Temporal and Activity-Related Programs. J. Neurosci 43, 28–55. 10.1523/jneurosci.0884-22.2022. 36446587 PMC9838713

[24] Kozik P, Francis RW, Seaman MNJ, and Robinson MS (2010). A Screen for Endocytic Motifs. Traffic 11, 843–855. 10.1111/j.1600-0854.2010.01056.x. 20214754 PMC2882754

[25] Traub LM, and Bonifacino JS (2013). Cargo Recognition in Clathrin-Mediated Endocytosis. Cold Spring Harb. Perspect. Biol 5, a016790. 10.1101/cshperspect.a016790. 24186068 PMC3809577

[26] Gillooly DJ, Morrow IC, Lindsay M, Gould R, Bryant NJ, Gaullier JM, Parton RG, and Stenmark H (2000). Localization of phosphatidylinositol 3-phosphate in yeast and mammalian cells. EMBO J. 19, 4577–4588. 10.1093/emboj/19.17.4577. 10970851 PMC302054

[27] Thompson A, Schäfer J, Kuhn K, Kienle S, Schwarz J, Schmidt G, Neumann T, Johnstone R, Mohammed AKA, and Hamon C (2003). Tandem Mass Tags: A Novel Quantification Strategy for Comparative Analysis of Complex Protein Mixtures by MS/MS. Anal. Chem 75, 1895–1904. 10.1021/ac0262560. 12713048

[28] Smith GA, Howell GJ, Phillips C, Muench SP, Ponnambalam S, and Harrison MA (2016). Extracellular and Luminal pH Regulation by Vacuolar H+-ATPase Isoform Expression and Targeting to the Plasma Membrane and Endosomes. J. Biol. Chem 291, 8500–8515. 10.1074/jbc.m116.723395. 26912656 PMC4861423

[29] Lin W-Y, Williams C, Yan C, Koledachkina T, Luedke K, Dalton J, Bloomsburg S, Morrison N, Duncan KE, Kim CC, (2015). The SLC36 transporter Pathetic is required for extreme dendrite growth in Drosophila sensory neurons. Genes Dev. 29, 1120–1135. 10.1101/gad.259119.115. 26063572 PMC4470281

[30] Hung V, Zou P, Rhee H-W, Udeshi ND, Cracan V, Svinkina T, Carr SA, Mootha VK, and Ting AY (2014). Proteomic Mapping of the Human Mitochondrial Intermembrane Space in Live Cells via Ratiometric APEX Tagging. Mol. Cell 55, 332–341. 10.1016/j.molcel.2014.06.003. 25002142 PMC4743503

[31] Zhou L, Xue X, Yang K, Feng Z, Liu M, and Pastor-Pareja JC (2022). Convergence of secretory, endosomal, and autophagic routes in trans-Golgi–associated lysosomes. J. Cell Biol 222, e202203045. 10.1083/jcb.202203045. 36239631 PMC9577102

[32] Toshima JY, and Toshima J (2024). Transport mechanisms between the endocytic, recycling, and biosynthetic pathways via endosomes and the trans-Golgi network. Front. Cell Dev. Biol 12, 1464337. 10.3389/fcell.2024.1464337. 39291266 PMC11405242

[33] Cullen PJ, and Steinberg F (2018). To degrade or not to degrade: mechanisms and significance of endocytic recycling. Nat. Rev. Mol. Cell Biol 19, 679–696. 10.1038/s41580-018-0053-7. 30194414

[34] Braulke T, and Bonifacino JS (2009). Sorting of lysosomal proteins. Biochim. Biophys. Acta 1793, 605–614. 10.1016/j.bbamcr.2008.10.016. 19046998

[35] Schwabl S, and Teis D (2022). Protein quality control at the Golgi. Curr. Opin. Cell Biol 75, 102074. 10.1016/j.ceb.2022.02.008. 35364487

[36] Fregno I, Fasana E, Bergmann TJ, Raimondi A, Loi M, Soldà T, Galli C, D’Antuono R, Morone D, Danieli A, (2018). ER-to-lysosome-associated degradation of proteasome-resistant ATZ polymers occurs via receptor-mediated vesicular transport. EMBO J. 37, e99259. 10.15252/embj.201899259. 30076131 PMC6120659

[37] Fregno I, Fasana E, Soldà T, Galli C, and Molinari M (2021). N-glycan processing selects ERAD-resistant misfolded proteins for ER-to-lysosome-associated degradation. EMBO J. 40, e107240. 10.15252/embj.2020107240. 34152647 PMC8327951

[38] Fasana E, Fregno I, Galli C, Soldà T, and Molinari M (2024). ER-to-lysosome-associated degradation acts as failsafe mechanism upon ERAD dysfunction. EMBO Rep. 25, 2773–2785. 10.1038/s44319-024-00165-y. 38773321 PMC11169228

[39] Roberts BS, Mitra D, Abishek S, Beher R, and Satpute-Krishnan P (2024). The p24-family and COPII subunit SEC24C facilitate the clearance of alpha1-antitrypsin Z from the endoplasmic reticulum to lysosomes. Mol. Biol. Cell 35, ar45. 10.1091/mbc.e23-06-0257. 38294851 PMC10916869

[40] Salomo-Coll C, Jimenez-Moreno N, and Wilkinson S (2025). Lysosomal degradation of ER client proteins by ER-phagy and related pathways. J. Mol. Biol 437, 169035. 10.1016/j.jmb.2025.169035. 39993592

[41] Hundley FV, Gonzalez-Lozano MA, Gottschalk LM, Cook ANK, Zhang J, Paulo JA, and Harper JW (2024). Endo-IP and lyso-IP toolkit for endolysosomal profiling of human-induced neurons. Proc. Natl. Acad. Sci. USA 121, e2419079121. 10.1073/pnas.2419079121. 39636867 PMC11670117

[42] Miyoshi J, and Takai Y (2008). Structural and functional associations of apical junctions with cytoskeleton. Biochim. Biophys. Acta 1778, 670–691. 10.1016/j.bbamem.2007.12.014. 18201548

[43] Babatz F, Naffin E, and Klämbt C (2018). The Drosophila Blood-Brain Barrier Adapts to Cell Growth by Unfolding of Pre-existing Septate Junctions. Dev. Cell 47, 697–710.e3. 10.1016/j.devcel.2018.10.002. 30482667

[44] Veeraval L, O’Leary CJ, and Cooper HM (2020). Adherens Junctions: Guardians of Cortical Development. Front. Cell Dev. Biol 8, 6. 10.3389/fcell.2020.00006. 32117958 PMC7025593

[45] Verkhratsky A, and Pivoriūnas A (2023). Astroglia support, regulate and reinforce brain barriers. Neurobiol. Dis 179, 106054. 10.1016/j.nbd.2023.106054. 36842485

[46] Muthukumar AK, Stork T, and Freeman MR (2014). Activity-dependent regulation of astrocyte GAT levels during synaptogenesis. Nat. Neurosci 17, 1340–1350. 10.1038/nn.3791. 25151265 PMC4176984

[47] Wu B, Li J, Chou Y-H, Luginbuhl D, and Luo L (2017). Fibroblast growth factor signaling instructs ensheathing glia wrapping of Drosophila olfactory glomeruli. Proc. Natl. Acad. Sci. USA 114, 7505–7512. 10.1073/pnas.1706533114. 28674010 PMC5530699

[48] Tepass U, Theres C, and Knust E (1990). crumbs encodes an EGF-like protein expressed on apical membranes of Drosophila epithelial cells and required for organization of epithelia. Cell 61, 787–799. 10.1016/0092-8674(90)90189-l. 2344615

[49] Bulgakova NA, and Knust E (2009). The Crumbs complex: from epithelial-cell polarity to retinal degeneration. J. Cell Sci 122, 2587–2596. 10.1242/jcs.023648. 19625503

[50] Tepass U (1996). Crumbs, a Component of the Apical Membrane, Is Required for Zonula Adherens Formation in Primary Epithelia of Drosophila. Dev. Biol 177, 217–225. 10.1006/dbio.1996.0157. 8660889

[51] Johnson K, Grawe F, Grzeschik N, and Knust E (2002). Drosophila Crumbs Is Required to Inhibit Light-Induced Photoreceptor Degeneration. Curr. Biol 12, 1675–1680. 10.1016/s0960-9822(02)01180-6. 12361571

[52] Bujakowska K, Audo I, Mohand-Saïd S, Lancelot ME, Antonio A, Germain A, Léveillard T, Letexier M, Saraiva JP, Lonjou C, (2012). CRB1 mutations in inherited retinal dystrophies. Hum. Mutat 33, 306–315. 10.1002/humu.21653. 22065545 PMC3293109

[53] den Hollander AI, Ghiani M, de Kok YJM, Wijnholds J, Ballabio A, Cremers FPM, and Broccoli V (2002). Isolation of Crb1, a mouse homologue of Drosophila crumbs, and analysis of its expression pattern in eye and brain. Mech. Dev 110, 203–207. 10.1016/s0925-4773(01)00568-8. 11744384

[54] Dolón JF, Paniagua AE, Valle V, Segurado A, Arévalo R, Velasco A, and Lillo C (2018). Expression and localization of the polarity protein CRB2 in adult mouse brain: a comparison with the CRB1rd8 mutant mouse model. Sci. Rep 8, 11652. 10.1038/s41598-018-30210-5. 30076417 PMC6076319

[55] Huang J, Zhou W, Dong W, Watson AM, and Hong Y (2009). Directed, efficient, and versatile modifications of the Drosophila genome by genomic engineering. Proc. Natl. Acad. Sci. USA 106, 8284–8289. 10.1073/pnas.0900641106. 19429710 PMC2688891

[56] Lee T, and Luo L (1999). Mosaic Analysis with a Repressible Cell Marker for Studies of Gene Function in Neuronal Morphogenesis. Neuron 22, 451–461. 10.1016/s0896-6273(00)80701-1. 10197526

[57] Tepass U, and Knust E (1993). crumbs and stardust Act in a Genetic Pathway That Controls the Organization of Epithelia in Drosophila melanogaster. Dev. Biol 159, 311–326. 10.1006/dbio.1993.1243. 8365569

[58] Simões S, Lerchbaumer G, Pellikka M, Giannatou P, Lam T, Kim D, Yu J, Ter Stal D, Al Kakouni K, Fernandez-Gonzalez R, (2022). Crumbs complex–directed apical membrane dynamics in epithelial cell ingression. J. Cell Biol 221, e202108076. 10.1083/jcb.202108076. 35588693 PMC9123285

[59] Cho NK, Keyes L, Johnson E, Heller J, Ryner L, Karim F, and Krasnow MA (2002). Developmental Control of Blood Cell Migration by the Drosophila VEGF Pathway. Cell 108, 865–876. 10.1016/s0092-8674(02)00676-1. 11955438

[60] Brückner K, Kockel L, Duchek P, Luque CM, Rørth P, and Perrimon N (2004). The PDGF/VEGF Receptor Controls Blood Cell Survival in Drosophila. Dev. Cell 7, 73–84. 10.1016/j.devcel.2004.06.007. 15239955

[61] Parsons B, and Foley E (2013). The Drosophila Platelet-derived Growth Factor and Vascular Endothelial Growth Factor-Receptor Related (Pvr) Protein Ligands Pvf2 and Pvf3 Control Hemocyte Viability and Invasive Migration. J. Biol. Chem 288, 20173–20183. 10.1074/jbc.m113.483818. 23737520 PMC3711285

[62] Wu L, Pandey V, Casha VH, Qu Z, Jami-Alahmadi Y, Gradinaru V, Wohlschlegel JA, and Khakh BS (2025). The cell-surface shared proteome of astrocytes and neurons and the molecular foundations of their multicellular interactions. Neuron 113, 2599–2620.e7. 10.1016/j.neuron.2025.05.019. 40499536 PMC12354340

[63] Takano T, Wallace JT, Baldwin KT, Purkey AM, Uezu A, Courtland JL, Soderblom EJ, Shimogori T, Maness PF, Eroglu C, (2020). Chemico-genetic discovery of astrocytic control of inhibition in vivo. Nature 588, 296–302. 10.1038/s41586-020-2926-0. 33177716 PMC8011649

[64] Ghoochani A, Heiby JC, Rawat ES, Medoh UN, Di Fraia D, Dong W, Gastou M, Nyame K, Laqtom NN, Gomez-Ospina N, (2024). Cell-Type Resolved Protein Atlas of Brain Lysosomes Identifies SLC45A1-Associated Disease as a Lysosomal Disorder. Preprint at bioRxiv. 10.1101/2024.10.14.618295. 41576950

[65] Yu Y, Gao SM, Guan Y, Hu P-W, Zhang Q, Liu J, Jing B, Zhao Q, Sabatini DM, Abu-Remaileh M, (2024). Organelle proteomic profiling reveals lysosomal heterogeneity in association with longevity. eLife 13, e85214. 10.7554/elife.85214. 38240316 PMC10876212

[66] Yap CC, and Winckler B (2015). Adapting for endocytosis: roles for endocytic sorting adaptors in directing neural development. Front. Cell. Neurosci 9, 119. 10.3389/fncel.2015.00119. 25904845 PMC4389405

[67] Smith CJ, O’Brien T, Chatzigeorgiou M, Spencer WC, Feingold-Link E, Husson SJ, Hori S, Mitani S, Gottschalk A, Schafer WR, (2013). Sensory Neuron Fates Are Distinguished by a Transcriptional Switch that Regulates Dendrite Branch Stabilization. Neuron 79, 266–280. 10.1016/j.neuron.2013.05.009. 23889932 PMC3795438

[68] Donatis AD, Comito G, Buricchi F, Vinci MC, Parenti A, Caselli A, Camici G, Manao G, Ramponi G, and Cirri P (2008). Proliferation Versus Migration in Platelet-derived Growth Factor Signaling THE KEY ROLE OF ENDOCYTOSIS*. J. Biol. Chem 283, 19948–19956. 10.1074/jbc.m709428200. 18499659

[69] Xu C, Li Z, Lyu C, Hu Y, McLaughlin CN, Wong KKL, Xie Q, Luginbuhl DJ, Li H, Udeshi ND, (2024). Molecular and cellular mechanisms of teneurin signaling in synaptic partner matching. Cell 187, 5081–5101.e19. 10.1016/j.cell.2024.06.022. 38996528 PMC11833509

[70] Qin W, Cho KF, Cavanagh PE, and Ting AY (2021). Deciphering molecular interactions by proximity labeling. Nat. Methods 18, 133–143. 10.1038/s41592-020-01010-5. 33432242 PMC10548357

[71] Li Y, Tian C, Liu K, Zhou Y, Yang J, and Zou P (2020). A Clickable APEX Probe for Proximity-Dependent Proteomic Profiling in Yeast. Cell Chem. Biol 27, 858–865.e8. 10.1016/j.chembiol.2020.05.006. 32470320

[72] Hall S, Bone C, Oshima K, Zhang L, McGraw M, Lucas B, Fehon RG, and Ward RE (2014). Macroglobulin complement-related encodes a protein required for septate junction organization and paracellular barrier function in Drosophila. Development 141, 889–898. 10.1242/dev.102152. 24496625 PMC3912832

[73] Golic KG, and Lindquist S (1989). The FLP recombinase of yeast catalyzes site-specific recombination in the drosophila genome. Cell 59, 499–509. 10.1016/0092-8674(89)90033-0. 2509077

[74] Xu T, and Rubin GM (1993). Analysis of genetic mosaics in developing and adult Drosophila tissues. Development 117, 1223–1237. 10.1242/dev.117.4.1223. 8404527

[75] Newsome TP, Åsling B, and Dickson BJ (2000). Analysis of Drosophila photoreceptor axon guidance in eye-specific mosaics. Development 127, 851–860. 10.1242/dev.127.4.851. 10648243

[76] Endo K, Aoki T, Yoda Y, Kimura K, and Hama C (2007). Notch signal organizes the Drosophila olfactory circuitry by diversifying the sensory neuronal lineages. Nat. Neurosci 10, 153–160. 10.1038/nn1832. 17220884

[77] Sweeney LB, Couto A, Chou Y-H, Berdnik D, Dickson BJ, Luo L, and Komiyama T (2007). Temporal Target Restriction of Olfactory Receptor Neurons by Semaphorin-1a/PlexinA-Mediated Axon-Axon Interactions. Neuron 53, 185–200. 10.1016/j.neuron.2006.12.022. 17224402

[78] Dietzl G, Chen D, Schnorrer F, Su K-C, Barinova Y, Fellner M, Gasser B, Kinsey K, Oppel S, Scheiblauer S, (2007). A genome-wide transgenic RNAi library for conditional gene inactivation in Drosophila. Nature 448, 151–156. 10.1038/nature05954. 17625558

[79] Pfeiffer BD, Truman JW, and Rubin GM (2012). Using translational enhancers to increase transgene expression in Drosophila. Proc. Natl. Acad. Sci. USA 109, 6626–6631. 10.1073/pnas.1204520109. 22493255 PMC3340069

[80] Aberle H, Haghighi AP, Fetter RD, McCabe BD, Magalhães TR, and Goodman CS (2002). wishful thinking Encodes a BMP Type II Receptor that Regulates Synaptic Growth in Drosophila. Neuron 33, 545–558. 10.1016/s0896-6273(02)00589-5. 11856529

[81] Potter CJ, Tasic B, Russler EV, Liang L, and Luo L (2010). The Q System: A Repressible Binary System for Transgene Expression, Lineage Tracing, and Mosaic Analysis. Cell 141, 536–548. 10.1016/j.cell.2010.02.025. 20434990 PMC2883883

[82] Hong W, Zhu H, Potter CJ, Barsh G, Kurusu M, Zinn K, and Luo L (2009). Leucine-rich repeat transmembrane proteins instruct discrete dendrite targeting in an olfactory map. Nat. Neurosci 12, 1542–1550. 10.1038/nn.2442. 19915565 PMC2826190

[83] Ito K, Suzuki K, Estes P, Ramaswami M, Yamamoto D, and Strausfeld NJ (1998). The Organization of Extrinsic Neurons and Their Implications in the Functional Roles of the Mushroom Bodies in Drosophila melanogaster Meigen. Learn. Mem 5, 52–77. 10.1101/lm.5.1.52. 10454372 PMC311240

[84] Zhu H, and Luo L (2004). Diverse Functions of N-Cadherin in Dendritic and Axonal Terminal Arborization of Olfactory Projection Neurons. Neuron 42, 63–75. 10.1016/s0896-6273(04)00142-4. 15066265

[85] Dionne H, Hibbard KL, Cavallaro A, Kao J-C, and Rubin GM (2018). Genetic Reagents for Making Split-GAL4 Lines in Drosophila. Genetics 209, 31–35. 10.1534/genetics.118.300682. 29535151 PMC5937193

[86] Ni J-Q, Zhou R, Czech B, Liu L-P, Holderbaum L, Yang-Zhou D, Shim H-S, Tao R, Handler D, Karpowicz P, (2011). A genome-scale shRNA resource for transgenic RNAi in Drosophila. Nat. Methods 8, 405–407. 10.1038/nmeth.1592. 21460824 PMC3489273

[87] Perkins LA, Holderbaum L, Tao R, Hu Y, Sopko R, McCall K, Yang-Zhou D, Flockhart I, Binari R, Shim H-S, (2015). The Transgenic RNAi Project at Harvard Medical School: Resources and Validation. Genetics 201, 843–852. 10.1534/genetics.115.180208. 26320097 PMC4649654

[88] Supek F, Bošnjak M, Škunca N, and Šmuc T (2011). REVIGO Summarizes and Visualizes Long Lists of Gene Ontology Terms. PLoS One 6, e21800. 10.1371/journal.pone.0021800. 21789182 PMC3138752

[89] Xie Q, Li J, Li H, Udeshi ND, Svinkina T, Orlin D, Kohani S, Guajardo R, Mani DR, Xu C, (2022). Transcription factor Acj6 controls dendrite targeting via a combinatorial cell-surface code. Neuron 110, 2299–2314.e8. 10.1016/j.neuron.2022.04.026. 35613619 PMC9308693

[90] Wu JS, and Luo L (2006). A protocol for dissecting Drosophila melanogaster brains for live imaging or immunostaining. Nat. Protoc 1, 2110–2115. 10.1038/nprot.2006.336. 17487202

[91] Charng W-L, Yamamoto S, Jaiswal M, Bayat V, Xiong B, Zhang K, Sandoval H, David G, Gibbs S, Lu H-C, (2014). Drosophila Tempura, a Novel Protein Prenyltransferase α Subunit, Regulates Notch Signaling Via Rab1 and Rab11. PLoS Biol. 12, e1001777. 10.1371/journal.pbio.1001777. 24492843 PMC3904817

[92] O’Brien CE, Younger SH, Jan LY, and Jan YN (2022). The GARP complex prevents sterol accumulation at the trans-Golgi network during dendrite remodeling. J. Cell Biol 222, e202112108. 10.1083/jcb.202112108. 36239632 PMC9577387

[93] Jay TR, Kang Y, Jefferson A, and Freeman MR (2021). An ELISA-based method for rapid genetic screens in Drosophila. Proc. Natl. Acad. Sci. USA 118, e2107427118. 10.1073/pnas.2107427118. 34686600 PMC8639337

[94] Lin C-C, and Potter CJ (2016). Editing Transgenic DNA Components by Inducible Gene Replacement in Drosophila melanogaster. Genetics 203, 1613–1628. 10.1534/genetics.116.191783. 27334272 PMC4981265

[95] Ludwig D, and Cable RM (1933). The Effect of Alternating Temperatures on the Pupal Development of Drosophila melanogaster Meigen. Physiol. Zoöl 6, 493–508. 10.1086/physzool.6.4.30151203.

[96] Park H, Hundley FV, Yu Q, Overmyer KA, Brademan DR, Serrano L, Paulo JA, Paoli JC, Swarup S, Coon JJ, (2022). Spatial snapshots of amyloid precursor protein intramembrane processing via early endosome proteomics. Nat. Commun 13, 6112. 10.1038/s41467-022-33881-x. 36245040 PMC9573879

[97] Hodge RD, Bakken TE, Miller JA, Smith KA, Barkan ER, Graybuck LT, Close JL, Long B, Johansen N, Penn O, (2019). Conserved cell types with divergent features in human versus mouse cortex. Nature 573, 61–68. 10.1038/s41586-019-1506-7. 31435019 PMC6919571

