# Supplementary material for: Endocytome profiling uncovers cell-surface protein dynamics underlying neuronal connectivity
Source: Neuron. Author manuscript; Available in PMC 2026 Apr 8. (PMC13055848; doi:10.1016/j.neuron.2026.01.027)
Supplement: MMC1 [file NIHMS2144613-supplement-MMC1.pdf]

Neuron, Volume *114*

Supplemental information

Endocytome profiling uncovers cell-surface protein dynamics underlying neuronal connectivity

Colleen N. McLaughlin, Hui Ji, Katherine X. Dong, Chuanyun Xu, Kenneth Kin Lam Wong, Zhuoran Li, David J. Luginbuhl, Charles Xu, Cheng Lyu, Wei Qin, Jiefu Li, Namrata D. Udeshi, Steven A. Carr, Alice Y. Ting, and Liqun Luo

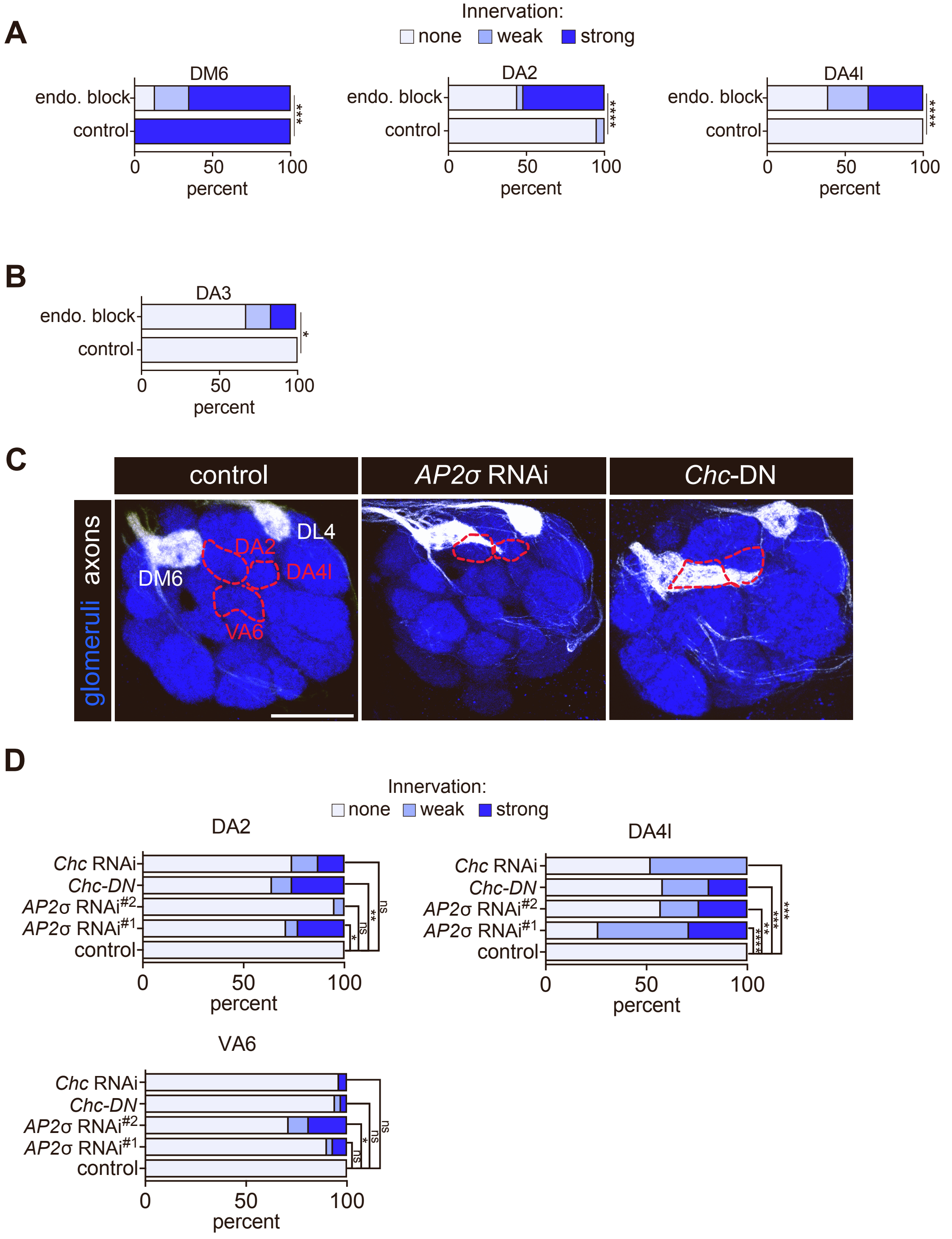

Figure S1. Clathrin-mediated endocytosis is critical for axon targeting, related to Figure 1.
(A, B) Quantification of axon targeting phenotype in DM6 (A) and DL4 (B) axons from Figure 1D, E. n = 23 (controls); n = 53 (endo. block).
(C) Images of axons innervating the DM6 and DL4 glomeruli (white, labeled with membrane tdTomato) of control (left) or those expressing UAS-RNAi against the σ-subunit of the AP2 complex (middle) or a dominant negative (DN) form of Clathrin heavy chain (Chc) (right) using the *AM29-GAL4* driver. Both AP2 and Chc mediate Cathrin-dependent endocytosis of CSPs. Brains from 48–72h APF pupae were used for these experiments because the RNAi lines are constitutively expressed, and dissecting at this time point enabled us to monitor axon targeting defects caused by

disrupting endocytosis during development rather than secondary effects in mature neurons. In addition, some lines caused adult lethality. Dotted outlines denote glomeruli where ectopic targeting is observed. Scale bar, 20 µm.
(D) Quantification of DM6 axon mistargeting phenotypes. n = 23 (control); n = 21 (*AP-2σ* RNAi$^{\#1}$); n = 31 (*AP-2σ* RNAi$^{\#2}$); n = 31 (Chc-DN); n = 23 (*Chc* RNAi).
Fisher's exact test was used to determine statistical significance.

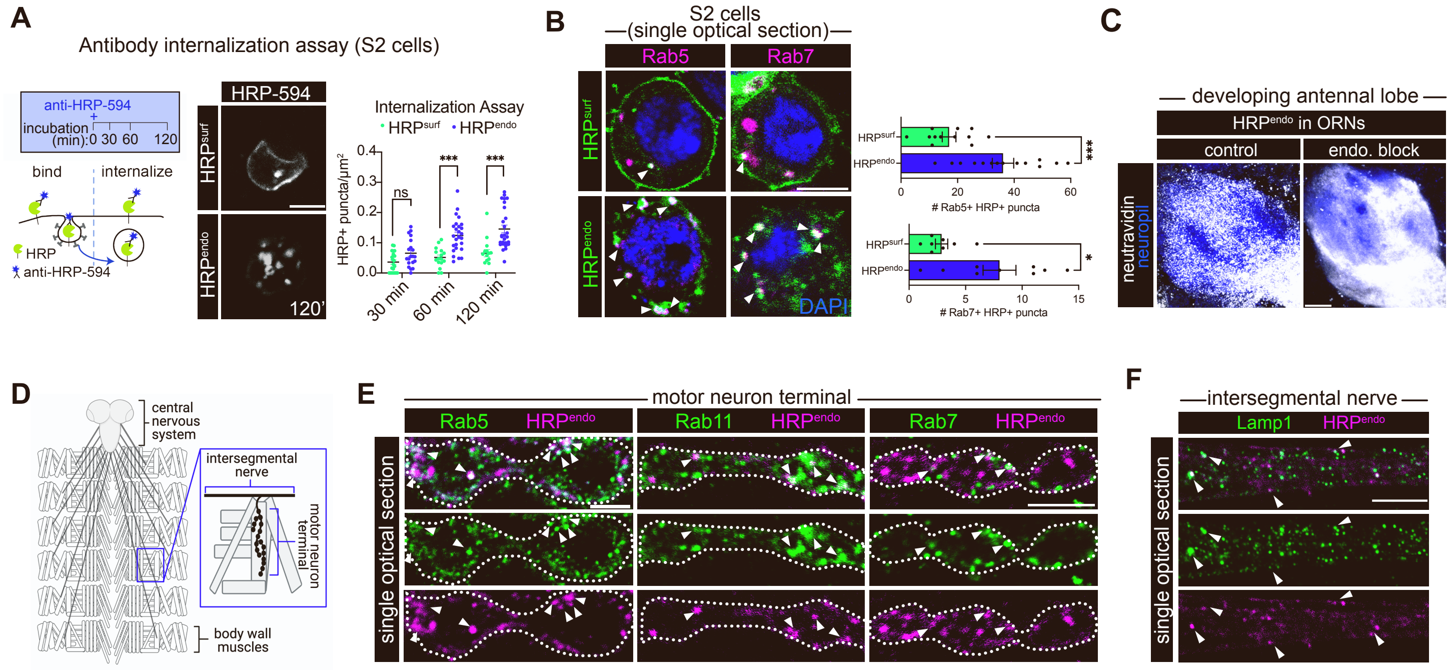

Figure S2. Validation of endosome-targeted HRP, related to Figure 1.

(A) Schematic of antibody internalization assay performed in S2 cells (left); single-optical section of S2 cells from internalization assay (middle); and quantification of the number of internalized HRP-positive puncta per area (right). Note that some HRP^{surf} is likely internalized at a low level due to its high surface expression. Number of cells for each experiment are: n = 22 (30 min), 16 (60 min), 15 (120 min) for HRP^{surf}; n = 20 (30 min), 26 (60 min), 27 (120 min) for HRP^{endo}.

(B) Images of S2 cells depicting co-labeling of indicated HRP and either Rab5 (early endosomes) or Rab7 (late endosomes) (left). Quantification of the number of HRP+ Rab+ puncta (right). Number of cells for each experiment are: n = 12 (Rab5), 8 (Rab7) for HRP^{surf}; n = 14 (Rab5), 9 (Rab7) for HRP^{endo}.

(C) Images depicting the redistribution of neutravidin labeling from endosomes (left) to the surface (right) of ORN axons when endocytosis is blocked from 0–36h APF using *Peb>UAS-shi^{ts}*.

(D) Schematic of the third instar larval central nervous system and body wall muscles. Inset depicts the neuromuscular junction (NMJ). Motor neuron terminals were used to test colocalization because it is a large synapse of a single neuron, and colocalization can be performed *in vivo* with antibodies against endogenous proteins rather than overexpressed Rab/lysosomal proteins.

(E) Images of motor neuron terminals (NMJs) depicting colocalization between HRP^{endo} and either Rab5, Rab11, or Rab7. Dotted outline denotes motor neuron membrane.

(F) Images of intersegmental nerves depicting colocalization between HRP^{endo} and Lamp1.

Mann-Whitney test was used to determine statistical significance. Error bars represent standard error of the mean (SEM). Anti-HA was used to detect HRP transgenes unless otherwise noted. Scale bar, 5 µm (A, B, E), 10 µm (C, F). Genotypes of all S2 cell experiments are either *UAS-HA-HRP^{surf}* or *UAS-HA-HRP^{endo}* driven by *Act-GAL4*. Arrowheads denote colocalization.

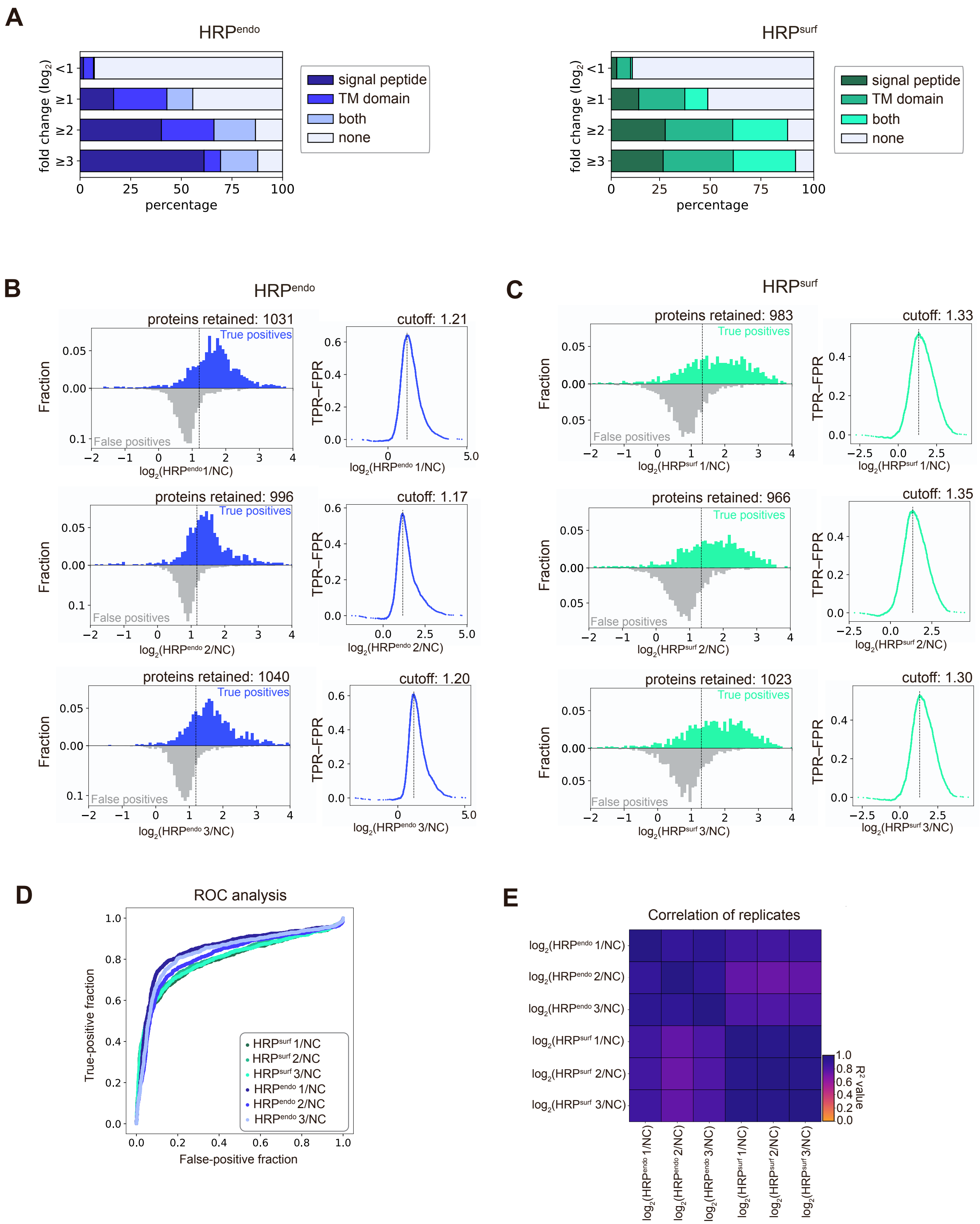

Figure S3. Analysis of ORN endocytomes and surfaceomes, related to Figure 2.
(A) Percentage of proteins labeled by HRP^{endo} (left) or HRP^{surf} (right) that contain a transmembrane (TM) domain or signal peptide (used to determine true-positive proteins). Fold change is compared to negative controls (NC).

(B, C) TMT-ratio cutoff in each biological replicate. Cutoffs were set to maximize true-positive rate – false-positive rate (TPR – FPR). True-positives are proteins with a signal peptide or TM domain and false-positives did not have these signatures and are annotated to the nucleus, mitochondria, or cytosol/cytoplasm by UniProt.
(D) Receiver operating characteristic (ROC) analysis for each biological replicate generated by plotting the proportion of true-positive proteins against the proportion of false-positive proteins rank ordered (from 0,0) by enrichment in each sample.
(E) Correlation matrix of biological replicates. Negative control (NC) data was averaged for this analysis.

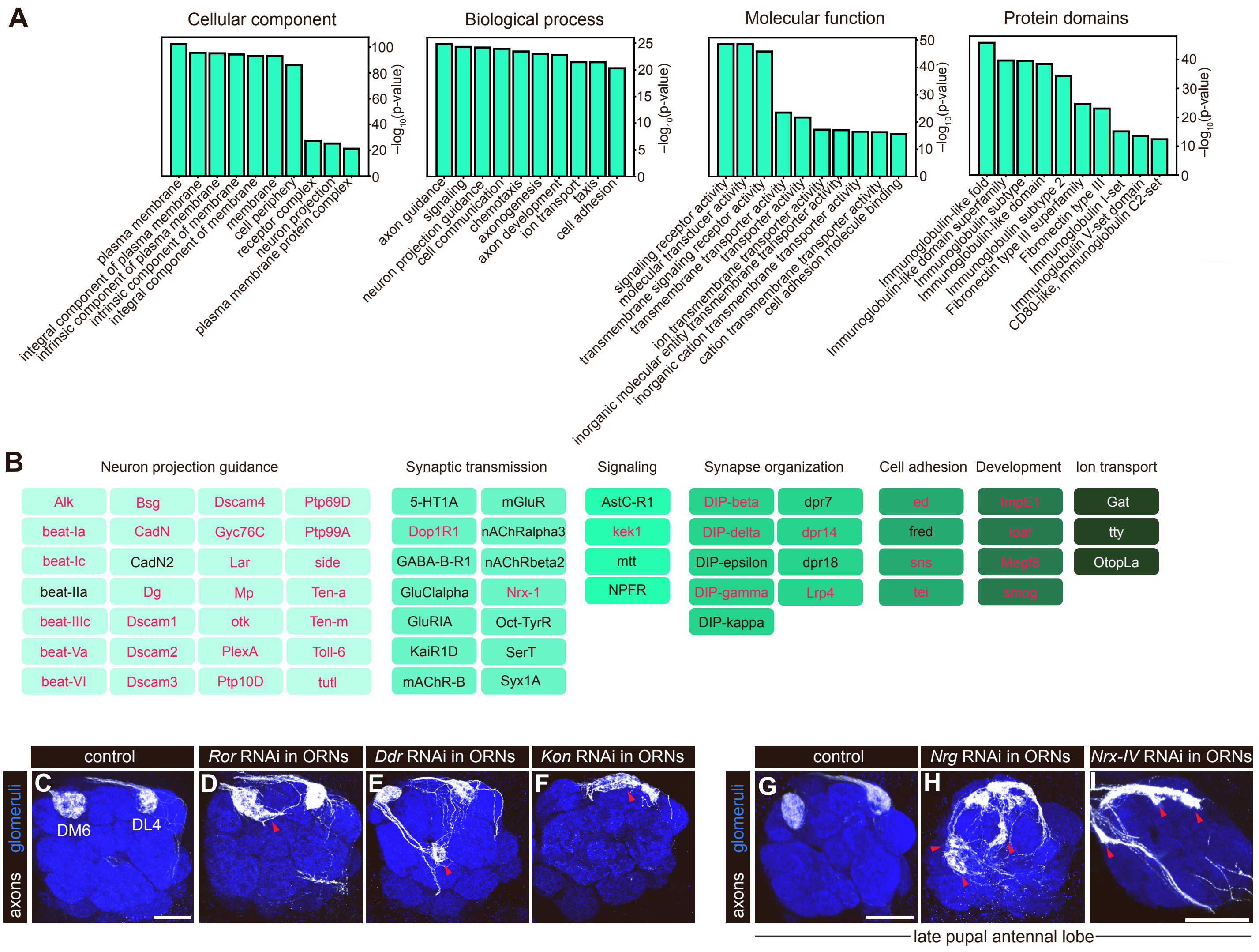

Figure S4. Additional characterization of surface-enriched membrane proteins, related to Figure 4.
(A) GO analysis of TM proteins present at the ORN axonal surface. Proteins with a $\log_2(\mathrm{HRP}^{\mathrm{surf}}/\mathrm{HRP}^{\mathrm{endo}}$ fold change) ≥ 0.1 were used for this analysis.
(B) 66 of the top 100 CSPs enriched in surfaceomes and the biological process they are most associated with. For simplicity, we prioritized displaying GO categories that had more than 2 proteins annotated to them. The complete list of proteins, including unannotated ones, can be found in Table S2. Pink text indicates that these proteins have known neurodevelopmental functions according to Flymine GO evidence codes.
(C–I) Images of antennal lobes from the loss-of-function screen where membrane proteins enriched in ORN surfaceomes were knocked-down in all ORNs (via *Peb-GAL4)* and axons projecting to the DM6 and DL4 glomerulus were labeled with *AM29-QF2* driven membrane-targeted tdTomato (*QUAS-mtdTomato*; white). Arrowheads indicate DM6-ORN axon mistargeting. Note for the two RNAi lines that caused lethality at the adult stage, we analyzed phenotypes in the late (~72–96h APF) pupal stages (G–I). Quantification of phenotypic penetrance is in Table S4.

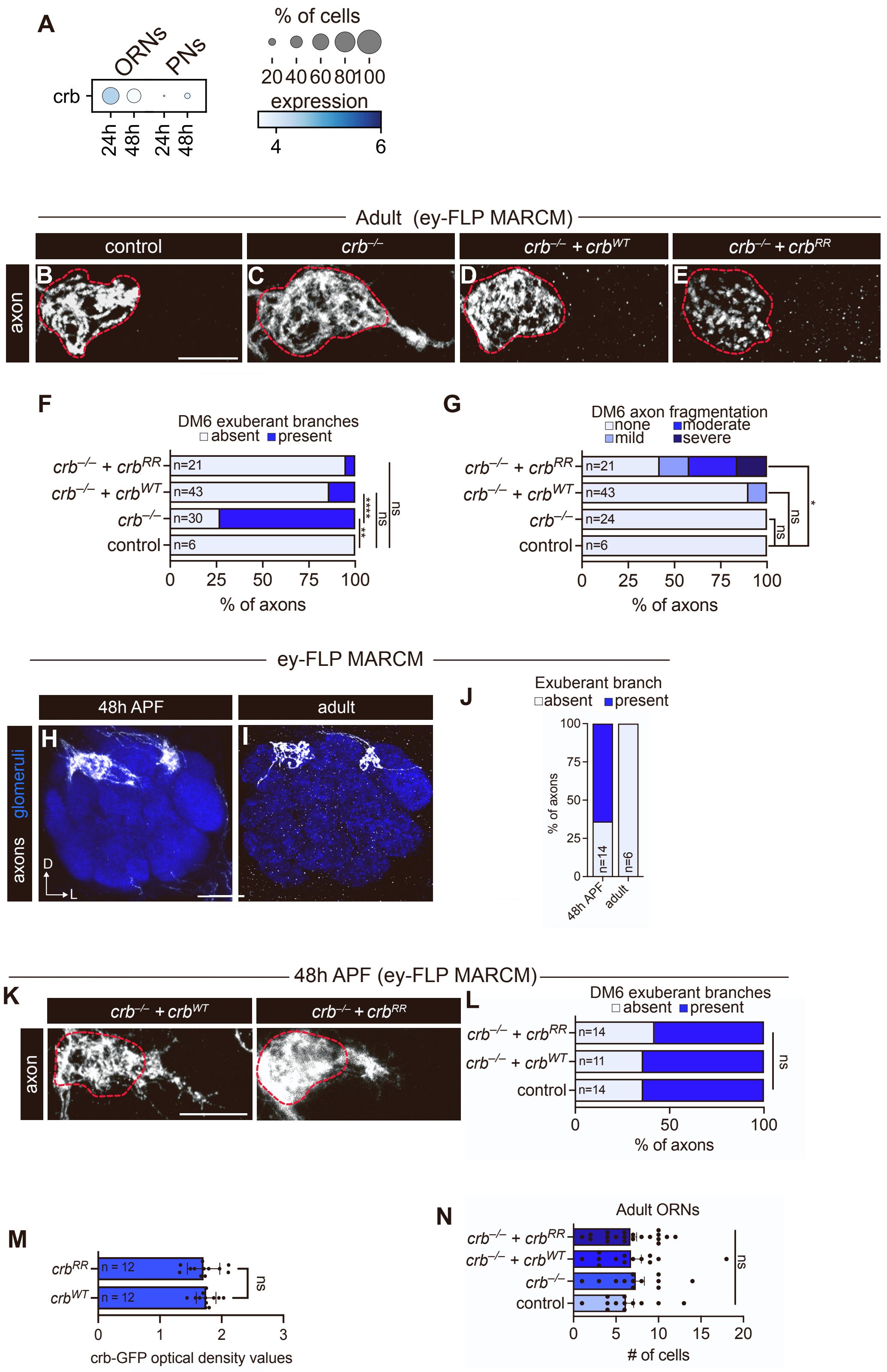

Figure S5. Additional MARCM-based mosaic analysis of *crb* mutant backgrounds, related to Figure 5.
(A) Dotplot depicting expression of *crb*. Expression unit is $\log_2$(CPM+1).
(B–E) Images of mosaic analysis using *ey-FLP* MARCM of DM6 axon phenotypes in indicated genotypes.
(F, G) Quantification of the percentage of DM6 axons that still have the exuberant branches present at the adult stage (F) or have axon fragmentation (G) in indicated genotypes.

(H, I) Images of *ey-FLP* DM6 axon clones during development (48h APF) and in the adult stage.
(J) Quantification of the number of antennal lobes where DM6 axons have exuberant branches at indicated time points.
(K) Images depicting exploring branches in DM6 axons of *ey-FLP* MARCM *crb* LOF clones that express either *crb* rescue construct, *UAS-GFP-crb*^{WT} (left) or *UAS-GFP-crb*^{RR} (right).
(L) Quantification of the number of antennal lobes where DM6 axons have exuberant branches in indicated genotypes.
(M) Quantification (based on GFP ELISA) of crb-GFP protein produced in the central nervous system from pan-neuronal expression of each *UAS-crb* transgene.
(N) Quantification of the number of cell bodies present in the adult antenna of *ey-FLP* MARCM clones in indicated genotypes. Number of antennae for each experiment are: n = 12 (control); 12 (*crb*$^{-/-}$); 14 (*crb*$^{-/-}$ + *crb*^{WT}); 20 (*crb*$^{-/-}$ + *crb*^{RR}).
Dashed lines denote DM6 glomerular boundary. Scale bar, 20 µm. Fisher's exact test (F, G, L), Mann-Whitney (M), and Kruskal-Wallis test (N) was used to determine statistical significance. Error bars represent standard error of the mean (SEM).

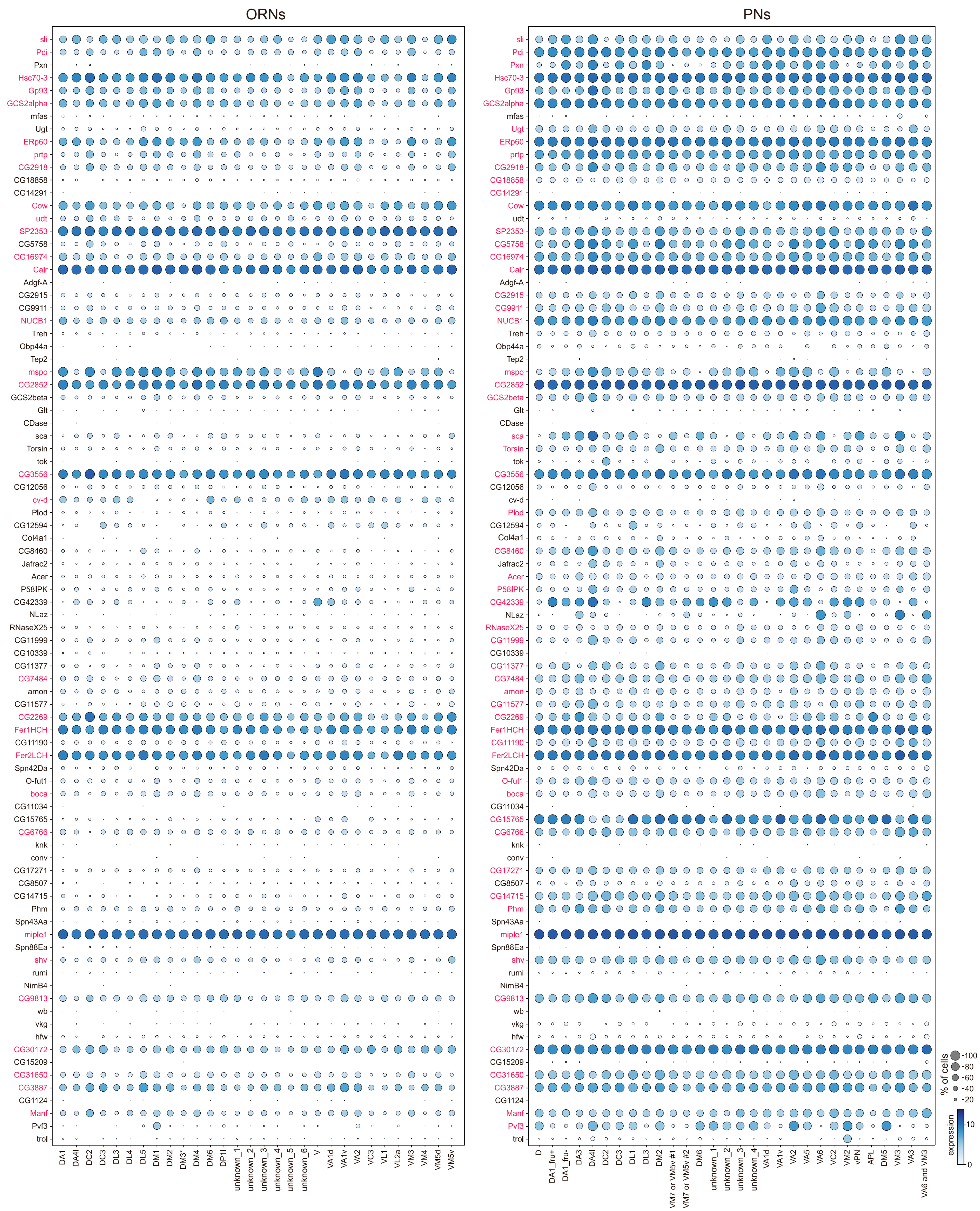
ORNs
PNs
sli
Pdi
Pxn
Hsc70-3
Gp93
GCS2alpha
mfas
Ugt
ERp60
prtp
CG2918
CG18858
CG14291
Cow
udt
SP2353
CG5758
CG16974
Calr
Adgf-A
CG2915
CG9911
NUCB1
Treh
Obp44a
Tep2
mspo
CG2852
GCS2beta
Glt
CDase
sca
Torsin
tok
CG3556
CG12056
cv-d
Plod
CG12594
Col4a1
CG8460
Jafrac2
Acer
P58IPK
CG42339
NLaz
RNaseX25
CG11999
CG10339
CG11377
CG7484
amon
CG11577
CG2269
Fer1HCH
CG11190
Fer2LCH
Spn42Da
O-fut1
boca
CG11034
CG15765
CG6766
knk
conv
CG17271
CG8507
CG14715
Phm
Spn43Aa
miple1
Spn88Ea
shv
rumi
NimB4
CG9813
wb
vkg
hfw
CG30172
CG15209
CG31650
CG3887
CG1124
Manf
Pvf3
trol
DA1
DA4l
DC2
DC3
DL3
DL4
DL5
DM1
DM2
DM3*
DM4
DM6
DP1l
unknown_1
unknown_2
unknown_3
unknown_4
unknown_5
unknown_6
V
VA1d
VA1v
VA2
VC3
VL1
VL2a
VM3
VM4
VM5d
VM5v
D
DA1_fru+
DA1_fru-
DA3
DA4l
DC2
DC3
DL1
DL3
DM2
VM7 or VM5v #1
VM7 or VM5v #2
DM6
unknown_1
unknown_2
unknown_3
unknown_4
VA1d
VA1v
VA2
VA5
VA6
VC2
VM2
vPN
APL
DM5
VM3
VA3
VA6 and VM3
% of cells
100
80
60
40
20
expression
10
0

Figure S6. Single-cell RNA-sequencing analysis of genes encoding endocytome-enriched secreted proteins, related to Figure 6. Dotplots depicting expression of transcripts encoding endocytome-enriched secreted proteins in ORNs (left) and PNs (right). Expression was averaged across two developmental timepoints (24h APF and 48h APF). Expression is in log_2(CPM + 1). Transcripts in pink met expression threshold (log_2[CPM+1] ≥ 4 in ≥ 30% of cells) and were retained for further analysis. 'unknown_#' denotes a cluster where the glomerular target of an ORN or PN subtype is unknown.
See Table S2 for additional data.

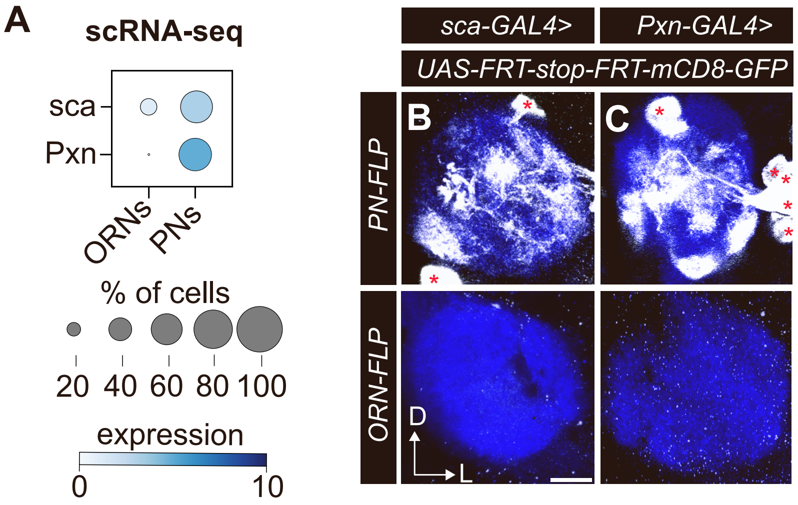

Figure S7. Depiction and validation of cell-type specific expression of endosome-enriched secreted proteins, Related to Figure 6.
(A) Dotplot depicting scRNA-seq data showing that *sca* and *Pxn* are preferentially expressed in PNs.
(B, C) Images of ~36h APF antennal lobes depicting GAL4 expression used to validate cell-type-specific expression found in Table S2. Asterisks denote PN cell bodies. Scale bar, 10 µm.

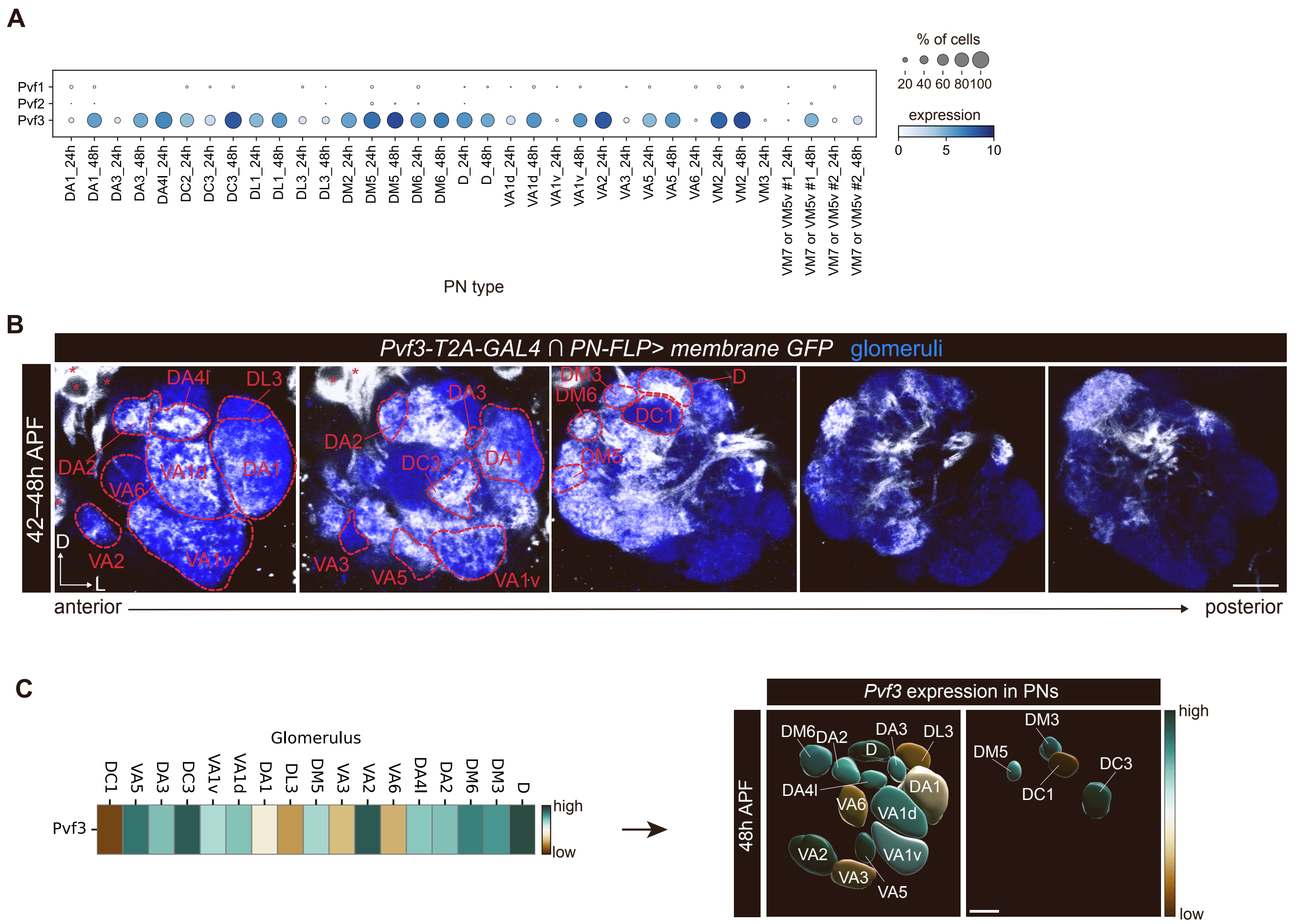

Figure S8. *Pvf3* expression analyses, related to Figure 7.
(A) Dotplot depicting expression of mRNAs (*Pvf1–3*) that encode Pvr's known ligands in 48–54h APF PNs. Expression unit is $\log_2$(CPM+1).
(B) Images along the anteroposterior axis of the developing antennal lobe of *Pvf3-T2A-GAL4* expression in PNs. Fluorescence intensity of encircled glomeruli was used to generate plots in Figure 7G. Asterisks denote PN cell bodies. Slight differences in transcript expression between (A) and (B) likely reflect differences in the stages where expression was analyzed (scRNA-seq is from 48-54h APF and fluorescence is from 42-48h APF). Scale bar, 10 µm.
(C) Heatmaps depicting averaged fluorescence intensity in each glomerulus of GAL4 lines in B. Fluorescence intensity in each glomerulus was normalized to the average intensity of all analyzed glomeruli. n ≥ 5 brains were analyzed for each GAL4 line. Heatmap colors were directly used to generate the overlay colors in Figure 7G. Expression is in arbitrary units (left). Volume rendering of a subset of surface glomeruli in the antennal lobe depicting *Pvf3* expression (right).

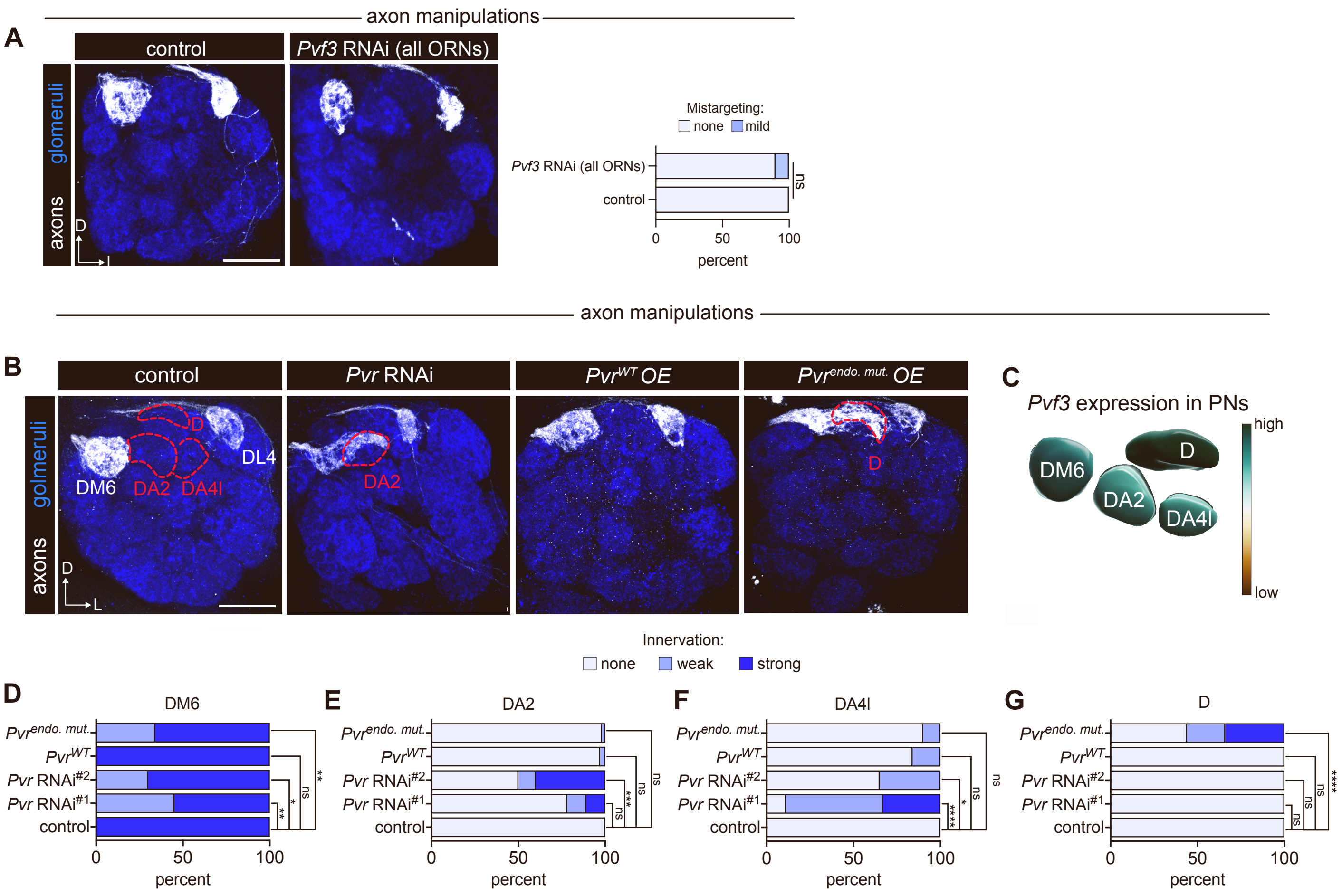

Figure S9. Extended analyses of axon targeting in *Pvf3* and *Pvr* loss- and gain-of-function manipulations in DM6 ORNs, related to Figure 7.

(A) Representative images of DM6-ORN and DL4-ORN axons of controls or when *Pvf3* is knocked down in all ORNs illustrating that there is no significant phenotype when the ligand is decreased in ORNs (left) and quantification of mistargeting in indicated genotypes (right). n = 19 (controls); n = 30 (*Pvf3* RNAi).

(B) Representative images of DM6-ORN axons in indicated genotypes. Encircled glomeruli denote where ectopic targeting occurs when *Pvr* is manipulated.

(C) Volume rendering depicting *Pvf3* expression in glomeruli analyzed in subsequent panels.

(D–G) Quantification of DM6-ORN axon mistargeting phenotypes. Fisher's exact test was used to determine statistical significance. n = 23 (controls); n = 18 (*Pvr RNAi*$^{\#1}$); n = 20 (*Pvr RNAi*$^{\#2}$); n = 31 (*Pvr*^{WT} OE); n = 41 (*Pvr*$^{endo.mut.}$ OE). Scale bar, 20 µm.

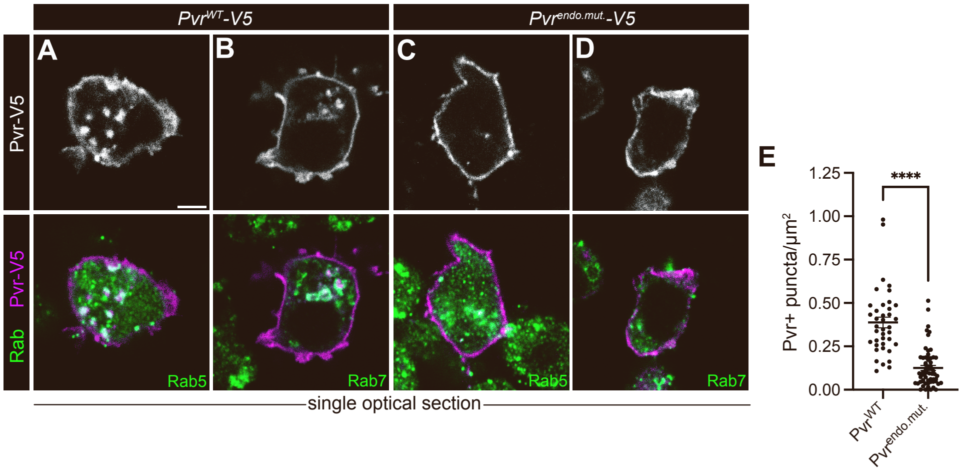

Figure S10. Extended analyses of Pvr overexpression transgenes, related to Figure 7.
(A–D) Images of S2 cells depicting co-labeling of indicated Pvr-V5 transgene and either Rab5 (A, C) or Rab7 (B, D).
(E) Quantification of the number of intracellular V5+ puncta per area. Number of intracellular puncta was normalized to the cell area to account for differences in cell size. Genotypes of experiments are either *UAS-Pvr*^{WT}*-V5* or *UAS-Pvr*$^{endo.mut.}$*-V5* driven by *Act-GAL4*. Number of cells for each experiment are: n = 39 (Pvr^{WT}), 61 (Pvr$^{endomut.}$). Kruskal-Wallis test was used to determine statistical significance. Error bars represent SEM. Scale bar is 2 µm.

Figure	Genotype	Notes
**Figure 1**		
**D,E**	*AM29-GAL4, UAS-S-mtdTomato-3xHA/+; 20XUAS-IVS-shits1-p10*_BDSC 66599	control = 18ºC; endo. Block = 24h @ 30ºC
**H**	*Peb-GAL4/+; UAS-HRP*^{endo}*/ UAS-mCherry-2xFYVE*	
**I**	*Peb-GAL4,UAS-myr-tdt/+; UAS-HRP*^{surf}*/+*	
**J**	*Peb-GAL4/+; UAS-HRP*^{endo}*/+*	
**K**	*Peb-GAL4/+; UAS-HRP*^{surf}*/+*	
**Figure 2**		
**A**	*Peb-GAL4/+; UAS-HRP*^{endo}*/+* (endosome)	
	Peb-GAL4/+; UAS-HRP^{surf}*/+* (surface)	
	w1118 (no enzyme control)	
	Peb-GAL4/+; UAS-HRP^{endo}*/+* (no hydrogen peroxide control)	
**Figure 3**		
**C**	*Peb-GAL4,UAS-myr-tdt/+*	
**D**	*Peb-GAL4/+; UAS-mCherry-2xFYVE/+*	
	Peb-GAL4/+; UAS-mCherry-2xFYVE/Gli-YFP	
**Figure 4**		
**D**	*Peb-GAL4, UAS-Dcr2/+;AM29-QF2, QUAS-mtdTomato-3xHA/+; UAS-luc*_BDSC 35788	
**E**	*Peb-GAL4, UAS-Dcr2/+;AM29-QF2, QUAS-mtdTomato-3xHA/+; UAS-bark RNAi*_VDRC 52608/+	
**F**	*Peb-GAL4, UAS-Dcr2/+;AM29-QF2, QUAS-mtdTomato-3xHA/+; UAS-crb RNAi*_BDSC 27697/+	
**G**	*Peb-GAL4, UAS-Dcr2/+;AM29-QF2, QUAS-mtdTomato-3xHA/UAS-Gli RNAi*_BDSC 38284	
**H**	*Peb-GAL4, UAS-Dcr2/+;AM29-QF2, QUAS-mtdTomato-3xHA/UAS-Tsf2 RNAi*_BDSC 65903	
**I**	*Peb-GAL4, UAS-Dcr2/+;AM29-QF2, QUAS-mtdTomato-3xHA/UAS-pck RNAi*_BDSC 38203	
**J**	*Peb-GAL4, UAS-Dcr2/+;AM29-QF2, QUAS-mtdTomato-3xHA/UAS-sinu RNAi*_VDRC 44928	
**Table S4**	*Peb-GAL4, UAS-Dcr2/+; AM29-QF2, QUAS-mtdTomato-3xHA/+ x* UAS-RNAi lines	Line numbers and stock centers are in table
**Figure 5**		
**B**	*Peb-GAL4; UAS-mCherry-2xFYVE/+; Crb-C-GFP/+*	
**C**	*hs-FLP, UAS-mCD8-GFP/+;AM29-GAL4,UAS-mtdTomato-3xHA/+; FRT82b, TubP-GAL80/FRT82b*	
**D**	*hs-FLP, UAS-mCD8-GFP/+;AM29-GAL4,UAS-mtdTomato-3xHA/+; FRT82b, TubP-GAL80/FRT82b, crb*^{11A22}	
**E**	*hs-FLP, UAS-mCD8-GFP/+;AM29-GAL4,UAS-mtdTomato-3xHA/+; FRT82b, TubP-GAL80/UAS-GFP-crb*^{WT}*,FRT82b, crb*^{11A22}	
**F**	*hs-FLP, UAS-mCD8-GFP/+;AM29-GAL4,UAS-mtdTomato-3xHA/+; FRT82b, TubP-GAL80/UAS-GFP-crb*^{RR}*,FRT82b, crb*^{11A22}	
**I**	*AM29-GAL4, UAS-mtdTomato-3xHA/+; UAS-GFP-crb*^{WT}*/+*	
**J**	*AM29-GAL4; UAS-mChery-2xFYVE/UAS-GFP-crb*^{WT}	
**K**	*AM29-GAL4, UAS-mtdTomato-3xHA/+; UAS-GFP-crb*^{RR}*/+*	
**L**	*AM29-GAL4; UAS-mChery-2xFYVE/UAS-GFP-crb*^{RR}	
**Figure 6**		
**G**	*AM29-QF2, QUAS-mtdTomato-3xHA/ UAS-luc; VT033006-Gal4/+*	BDSC_35788

H	AM29-QF2, QUAS-mtdTomato-3xHA/; VT033006-Gal4/UAS-shv-RNAi_VDRC 22997	BDSC_54797 (#1)<br>VDRC_22997 (#2)
I	AM29-QF2, QUAS-mtdTomato-3xHA/ UAS-Pvf3-RNAi_VDRC 105008; VT033006-Gal4/+	VDRC_105008 (#1)<br>BDSC_38962 (#2)
**Figure 7**		
B, C	UAS-dcr2, UAS-mCD8-GFP/+; Mz19-GAL4, UAS-mCD8-GFP, Or88a-mtdTomato/+; Or47b-rCD2/UAS-luc_BDSC 35788	
D	UAS-dcr2, UAS-mCD8-GFP/+; Mz19-GAL4, UAS-mCD8-GFP, Or88a-mtdTomato/UAS-Pvf3-RNAi_VDRC 105008; Or47b-rCD2/+	VDRC_105008 (#1)<br>BDSC_38962 (#2)
E	UAS-dcr2, UAS-mCD8-GFP/+; Mz19-GAL4, UAS-mCD8-GFP, Or88a-mtdTomato/+; Or47b-rCD2/UAS_Pvf3-myc	
H	UAS-dcr2, UAS-mCD8-GFP/+; R20D10-QF2, QUAS-mtdTomato-3xHA/+; R78H05-p65AD, R31F09-GAL4DBD/UAS-luc_BDSC 35788	Did not display labeling for R20D10>mtdTomato in I–L
I	UAS-dcr2, UAS-mCD8-GFP/+; R20D10-QF2, QUAS-mtdTomato-3xHA/UAS-Pvr RNAi_VDRC 977	VDRC_977 (#1); BDSC_37520 (#2)
J	UAS-dcr2, UAS-mCD8-GFP/+; R20D10-QF2, QUAS-mtdTomato-3xHA/+; R78H05-p65AD, R31F09-GAL4DBD/UAS-Pvr^WT-V5	
K	UAS-dcr2, UAS-mCD8-GFP/+; R20D10-QF2, QUAS-mtdTomato-3xHA/+; R78H05-p65AD, R31F09-GAL4DBD/UAS-Pvr $^{endo.mut}$.-V5	
Q	AM29-GAL4, UAS-mtdTomato-3xHA/+; UAS-Pvr^{WT}-V5/+	
R	AM29-GAL4, UAS-mCherry-2xFYVE/+; UAS-Pvr^{WT}-V5/+	
S	AM29-GAL4, UAS-mtdTomato-3xHA/+; UAS-Pvr$^{endo.mut.}$-V5/+	
T	AM29-GAL4, UAS-mCherry-2xFYVE/+; UAS-Pvr$^{endo.mut.}$-V5/+	
**Figure S1**		
C	AM29-GAL4, UAS-mtdTomato-3xHA/+; UAS-luc_BDSC 35788/+ (control)	
	AM29-GAL4, UAS-mtdTomato-3xHA/+; UAS-AP-2sigma_BSDC 27322/+	BSDC 27322 (#1); VDRC 110725 (#2)
	AM29-GAL4, UAS-mtdTomato-3xHA/+; UAS-Chc-DN_BDSC 26874/+	UAS-Chc RNAi = BSDC 34742
**Figure S2**		
C	Peb-GAL4, ey-FLP/+; UAS-FRT-stop-FRT-shi(ts1)/UAS-HRP^{endo}; UAS-FRT-stop-FRT-shi(ts1)/+	control = 18ºC; endo. block = 30ºC
E, F	OK6-GAL4/UAS-HRP^{endo}	
**Figure S4**		
C	Peb-GAL4, UAS-Dcr2/+;AM29-QF2, QUAS-mtdTomato-3xHA/+; UAS-luc_BDSC 35788	
D	Peb-GAL4, UAS-Dcr2/+;AM29-QF2, QUAS-mtdTomato-3xHA/UAS-Ror RNAi_BDSC 62868	
E	Peb-GAL4, UAS-Dcr2/+;AM29-QF2, QUAS-mtdTomato-3xHA/+; UAS-Ddr RNAi_VDRC 51719/+	
F	Peb-GAL4, UAS-Dcr2/+;AM29-QF2, QUAS-mtdTomato-3xHA/+; UAS-Kon RNAi_BDSC 31584/+	
G	Peb-GAL4, UAS-Dcr2/+;AM29-QF2, QUAS-mtdTomato-3xHA/+; UAS-luc_BDSC 35788	
H	Peb-GAL4, UAS-Dcr2/+;AM29-QF2, QUAS-mtdTomato-3xHA/UAS-Nrg RNAi_BDSC 38215	
I	Peb-GAL4, UAS-Dcr2/+; AM29-QF2, QUAS-mtdTomato-3xHA/+; UAS-Nrx-IV RNAi_BDSC 32424	
**Figure S5**		
B	ey-FLP, UAS-mCD8-GFP/+; AM29-GAL4, UAS-mtdTomato-3xHA/+; FRT82b, TubP-GAL80/FRT82b	
C	ey-FLP, UAS-mCD8-GFP/+; AM29-GAL4, UAS-mtdTomato-3xHA/+; FRT82b, TubP-GAL80/FRT82b, crb^{11A22}	
D	ey-FLP, UAS-mCD8-GFP/+; AM29-GAL4, UAS-mtdTomato-3xHA/+; FRT82b, TubP-GAL80/UAS-GFP-crb^{WT},FRT82b, crb^{11A22}	
E	ey-FLP, UAS-mCD8-GFP/+; AM29-GAL4, UAS-mtdTomato-3xHA/+; FRT82b, TubP-GAL80/UAS-GFP-crb^{RR},FRT82b, crb^{11A22}	
H, I	ey-FLP, UAS-mCD8-GFP/+; AM29-GAL4, UAS-mtdTomato-3xHA/+; FRT82b, TubP-GAL80/FRT82b	

K	*ey-FLP, UAS-mCD8-GFP/+; AM29-GAL4, UAS-mtdTomato-3xHA/+; FRT82b, TubP-GAL80/UAS-GFP-crb^{WT},FRT82b, crb^{11A22}* (left)	
	ey-FLP, UAS-mCD8-GFP/+; AM29-GAL4, UAS-mtdTomato-3xHA/+; FRT82b, TubP-GAL80/UAS-GFP-crb^{RR},FRT82b, crb^{11A22} (right)	
M	*elavGal4$^{4.1}$, elavGal4$^{4.4}$/ UAS-crb^{WT}*<br>*elavGal4$^{4.1}$, elavGal4$^{4.4}$/ UAS-crb^{RR}*	
N	*ey-FLP, UAS-mCD8-GFP/+; AM29-GAL4, UAS-mtdTomato-3xHA/+; FRT82b, TubP-GAL80/FRT82b*<br><br>*ey-FLP, UAS-mCD8-GFP/+; AM29-GAL4, UAS-mtdTomato-3xHA/+; FRT82b, TubP-GAL80/FRT82b, crb^{11A22}*<br><br>*ey-FLP, UAS-mCD8-GFP/+; AM29-GAL4, UAS-mtdTomato-3xHA/+; FRT82b, TubP-GAL80/UAS-GFP-crb^{WT},FRT82b, crb^{11A22}*<br><br>*ey-FLP, UAS-mCD8-GFP/+; AM29-GAL4, UAS-mtdTomato-3xHA/+; FRT82b, TubP-GAL80/UAS-GFP-crb^{RR},FRT82b, crb^{11A22}*	
**Figure S7**		
B	*GH146-FLP, UAS-FRT-stop-FRT-mCD8-GFP/sca-GAL4*_BDSC 6479 (top)	
	*ey-FLP3.5/+; UAS-FRT-stop-FRT-mCD8-GFP/sca-GAL4*_BDSC 6479 (bottom)	
C	*GH146-FLP, UAS-FRT-stop-FRT-mCD8-GFP/+; Pxn-GAL4/+*_BDSC 66850 (top)	
	*ey-FLP3.5/+; UAS-FRT-stop-FRT-mCD8-GFP/+; Pxn-GAL4/+*_BDSC 66850 (bottom)	
**Figure S8**		
B	*GH146-FLP, UAS-FRT-stop-FRT-mCD8-GFP/Pvf3-T2A-GAL4*	
**Figure S9**		
A	*Peb-GAL4, UAS-Dcr2/+; AM29-QF2, QUAS-mtdTomato-3xHA/+; UAS-luc*_BDSC 35788 (left)	
	*Peb-GAL4, UAS-Dcr2/+; AM29-QF2, QUAS-mtdTomato-3xHA/UAS-Pvf3-RNAi*_VDRC 105008 (right)	
B	*AM29-GAL4, UAS-mtdTomato-3xHA/+; UAS-luc*_BDSC 35788/+ (control)	*Pvr RNAi*_BDSC 37520 (#2)
	*AM29-GAL4, UAS-mtdTomato-3xHA/+; UAS-Pvr RNAi*_VDRC 977 (#1)	
	AM29-GAL4, UAS-mtdTomato-3xHA/UAS-Pvr^{WT}-V5	
	AM29-GAL4, UAS-mtdTomato-3xHA/UAS-Pvr$^{endo.mut.}$-V5	

Table S1. Complete list of genotypes used in this study.

Protein	Enrichment	Human ortholog(s)	DM6-ORN axon targeting deficits caused by RNAi knockdown	% Phenotypic penetrance (n)	RNAi lines
**bark**	endosome	–	axon trajectory error	27 (22); 56 (16)	BDSC 67014; VDRC 52608
**crb**	endosome	CRB1, CRB2	local mistargeting	20 (30); 74 (23)	BDSC 34999; BDSC 27697
**Gli**	endosome	NLGN1–3	local mistargeting	73 (33); 68 (31)	BDSC 38284; BDSC 58115
**pck**	endosome	Claudin	long-range mistargeting	79 (24); 19 (16)	BDSC 38203; VDRC50306
**sinu**	endosome	Claudin	long-range mistargeting	60 (30); 88 (17)	BDSC 38200; VDRC 44928
**Tsf2**	endosome	MELTF	local mistargeting	61 (28)	BDSC 65903
**Ddr**	surface	DDR1, DDR2	local and long-range mistargeting	31 (39); 81 (16)	VDRC 51719; BDSC 55906
**Kon**	surface	CSPG4	local mistargeting	64 (36); 52 (27)	BDSC 31583; BDSC 80477
**Nrg**	surface	NRCAM, L1CAM, CHL1, NFASC	local and long-range mistargeting; DM6 & DL4 merge; loss of bilateral innervation*	98 (40)	BDSC 38215
**Nrx-IV**	surface	CNTNAP1–3	loss of glomerular and terminal morphology; DM6 & DL4 terminals merge	79 (24); 77 (26)	BDSC 32424; VDRC 108128
**Ror**	surface	ROR1, ROR2	local mistargeting	73 (46); 41 (37)	BDSC 62868; VDRC 29930
**Drl-2**	surface	RYK	none	–	BDSC 55983; VDRC 40484

Table S4, related to Figure 4 and S4. A subset of endosome- and surface-enriched proteins were knocked-down in all ORNs (via *peb-GAL4)* and targeting to the DM6 glomerulus (using *AM29QF2, QUAS-mtdTomato*) was evaluated. Human orthologs were identified using Flybase Homologs search tool. The most predominant DM6 axon phenotype(s) is described with the penetrance of each RNAi line listed and the number of antennal lobes examined in parentheses. Note that *peb-GAL4* is expressed in tissues outside of the nervous system and when used to drive certain RNAi lines caused lethality, thus two proteins have a single RNAi line used for analysis. *Indicates that the loss of bilateral innervation caused by Nrg RNAi was previously described[S1].

References

[S1] Kaur R., Surala M., Hoger S., Grössmann N., Grimm A., Timaeus L., Kallina W., Hummel T. Pioneer interneurons instruct bilaterality in the Drosophila olfactory sensory map. Sci. Adv. 2019;5:eaaw5537. doi:10.1126/sciadv.aaw5537.
